# Bilingualism in Context: A Bayesian Psychometric Network Analysis of Language and Culture Among U.S. Heritage Spanish–English Speakers of Latin American Descent

**DOI:** 10.3390/bs16040522

**Published:** 2026-04-01

**Authors:** William Rayo, Ivan Carbajal

**Affiliations:** 1 Department of Psychology, University of California, Davis, CA 95616, USA; 2 School of Psychological Science, Oregon State University, Corvallis, OR 97331, USA; ivan.carbajal@oregonstate.edu

**Keywords:** bilingualism, biculturalism, heritage bilinguals, naturalistic language use, psychometric networks, bayesian statistics, attentional control

## Abstract

Bilingualism has increasingly been understood as a multidimensional and context-sensitive experience, prompting growing interest in how specific aspects of bilingual language use relate to cognition. We used Bayesian psychometric network analysis to examine how bilingual language practices, bicultural identity management, and cognition relate within the same system in a sample of 404 U.S.-born heritage Spanish–English bilingual adults of Latin American descent. This approach conceptualizes bilingualism as a complex system, quantifies uncertainty in the estimated network structure, and identifies aspects of bilingual experience that serve as bridges to cognition and bicultural identity. The strongest bridges between domains were the edge between language mixing and attentional control and the edge between unintended language switching and bicultural harmony. These findings provide a more holistic and socially infused characterization of how bilingualism, biculturalism, and cognition interact in U.S. heritage speakers of Spanish.

## 1. Introduction

Emergentist accounts of cognition and language (Bates, 1976; Bronfenbrenner, 1977) posit that our understanding of the world is built directly from our experiences. In their Bioecological Model, Bronfenbrenner and Ceci (1994) propose that human development is primarily shaped through consistent interactions with one’s immediate environment. These interactions, especially when they occur regularly and over long periods of time, play a key role in driving the development of experience-based changes in the brain. In this account, the timing of these interactions is critical, with those occurring earlier in life having greater potential to affect future development.

Language serves as a prime example of such an impactful experience; it permeates nearly all aspects of human interaction. Language, as a complex system of symbols and meanings, not only enables communication but also serves as a mechanism for passing on cultural values and shaping cognitive processes (Amici et al., 2019; Perlovsky, 2011). Empirical research supports the notion that language experiences impact neural and cognitive processes (Bice et al., 2020; Pliatsikas et al., 2020).

Bilingualism, in particular, provides a compelling case study for this idea. From the earliest stages of development, language acts as a potent catalyst for cognitive and neural restructuring (Bice & Kroll, 2019; D’Souza et al., 2020). Acquiring multiple languages (Byers-Heinlein et al., 2010), managing those languages (Byers-Heinlein et al., 2017), and progressing through to semantic organization (Borodkin et al., 2016) all exemplify the kind of dynamic and reciprocal interaction between the individual and the environment that the Bioecological Model identifies as a critical force in shaping human development.

### 1.1. Relationship Between the Mind, Brain, and Bilingualism

Studies have revealed that bilingual individuals activate all of their languages regardless of which one is being used at a particular moment (Kroll et al., 2012; Thierry & Wu, 2007). Moreover, switching from one language to another recruits the same neural and cognitive circuitry responsible for switching between tasks (De Baene et al., 2015; Declerck et al., 2017). Bilingualism not only modifies the structural and functional connectivity of the brain (Pliatsikas, 2019), but those changes are modulated by a variety of different language experiences (Gullifer et al., 2018; Hayakawa & Marian, 2019; X. Li et al., 2021; Pliatsikas et al., 2020).

Earlier work investigating the effects of bilingualism focused on identifying the existence of a “bilingual advantage” in which bilinguals outperform monolinguals across a variety of cognitive domains. However, this claim has been the subject of substantial debate, with replication efforts often finding small or mixed results (Antoniou, 2019; K. R. Paap et al., 2015). In response to these concerns, the field has increasingly shifted away from asking whether a bilingual advantage exists in a broad and uniform sense, and toward asking under what conditions particular bilingual experiences may be associated with differences in cognition (Bialystok & Craik, 2022; de Bruin, 2019; Woumans & Duyck, 2015). Critiques of the bilingual advantage hypothesis (Cespón, 2021; K. Paap, 2022) have played an important role in pushing the field toward greater methodological rigor (Beatty-Martínez & Titone, 2021; Blanco-Elorrieta & Pylkkänen, 2018; Ware et al., 2020). They have also encouraged researchers to reconceptualize bilingualism as a dynamic process shaped by ongoing interactions between individuals and their sociolinguistic environments (Filipović & Hawkins, 2019; Leivada et al., 2023; Titone & Tiv, 2022). Taken together, this debate has shifted the field toward a more nuanced account of the effects of bilingualism, emphasizing that they are heterogeneous and context-dependent rather than uniformly advantageous.

Bilinguals make up more than half of the world’s population, and as such, they differ in the languages they speak, the way they acquired those languages, and the contexts in which they use them (Luk & Bialystok, 2013). These variations in bilingual experiences are hypothesized to differentially impact the cognitive effects of using two languages, since adapting to varying environmental demands would result in distinct cognitive adaptations across bilingual individuals (Bialystok & Craik, 2022; Green, 2018; Green & Abutalebi, 2013). These theoretical accounts of the cognitive mechanisms involved in managing multiple languages emphasize the need for careful comparisons between different groups of bilinguals who vary on key variables of interest in order to identify the specific conditions under which bilingualism may influence cognitive functions (de Bruin, 2019; Woumans & Duyck, 2015). Modeling the impact of language and culture on cognition requires researchers to consider the language experiences of an individual situated within the social context in which they occurred. Recent theoretical and computational advances have enabled the use of dynamic systems theory to model bilingualism as an emergent phenomenon that arises from complex and direct interactions between individuals with unique language experiences and abilities adapting to the dynamic sociolinguistic demands of various environments over the course of their lives (Titone & Tiv, 2022).

### 1.2. Language Is Not a Solo Endeavor

Modeling language use as an exclusive interaction between the language-specific and domain-general components of the mind overlooks the intricate social fabric within which language operates. In fact, studies commonly exploit this relationship by using images of other people (Zhu et al., 2022), the presence of nonverbal participants (Bennani et al., 2023), or actual interlocutors (Kałamała et al., 2021) to experimentally manipulate performance on language tasks. Research on bilingualism requires careful consideration of conversational partners in addition to the social connotations that language choices carry.

Muysken (2013) proposes that the decision to use one or more languages in conversation is a bilingual optimization problem that is contingent upon three interrelated types of factors, language-specific factors, person-specific factors, and sociocultural factors. Language-specific factors such as the perceived distance between languages refer to the characteristics of the languages themselves. Languages that are closely related (e.g., Spanish and Italian) may have more overlapping sounds, vocabulary, grammatical structures, or ways of expressing ideas, making certain types of code-switching easier and more frequent. Person-specific factors refer to the bilingual’s language processing capabilities which influence the accessibility of lexical and syntactic structures. This construct is indexed via measures such as order of acquisition, language proficiency, or language dominance (de Bruin, 2019; Marian & Hayakawa, 2021; Rayo et al., 2024). Muysken (2013) proposes that bilingual individuals optimize their language use to minimize cognitive effort which results in selective deployment of code-switching, borrowing, or avoidance of certain linguistic structures that are more challenging to process. The critical final factor that needs consideration is the social context in which these language decisions are being made.

The choice and usage of languages are influenced by several social factors, including the status and prestige associated with different languages, societal attitudes towards bilingualism, and the specific communicative needs of a particular social context (Muysken, 2013). Code-switching practices vary widely, with opportunistic switches within sentences being common in metropolitan bilingual communities (Poplack, 1988; Tiv et al., 2022), to strategic utilization of code-switching for emphasis in others (Myslín & Levy, 2015), to being influenced by cultural norms and the sociopolitical valence of language choices (Muysken, 2000).

The variability in bilingual code-switching practices across sociolinguistic contexts found in Muysken (2013) underscores the need for psychological researchers to integrate the social dimensions of language use. Studies such as those by Beatty-Martínez et al. (2020) illustrate this complexity by comparing English–Spanish bilinguals across distinct sociolinguistic environments (Puerto Rico, Spain, and the United States), revealing how the distinct sociolinguistic norms regarding code-switching and bilingualism in each of these contexts resulted in varied engagement of distinct cognitive control mechanisms. Critically, traditional approaches focusing exclusively on individual language variables would have considered all of the participants part of the same English–Spanish speaking bilingual population and found null results. Overcoming these kinds of oversights requires a paradigm shift in psycholinguistics that incorporates the social dimensions of language use. Doing so promises us a more comprehensive understanding of bilingualism, one that considers the social nature of language use as a pivotal force in shaping both cognitive and linguistic development.

### 1.3. The Role of Culture

Vygotsky (1978) names the sociocultural environment as a fundamental force in shaping cognitive development, shaping not only what we learn but how we think. Culture and language go hand in hand; many bilingual individuals face the challenge of navigating not only multiple languages but also the distinct social norms and expectations inherent to various cultures. This phenomenon is highlighted in the work of West et al. (2017), which points out the growing necessity for people to adeptly manage cultural diversity alongside linguistic capabilities. Some scholars have suggested that it is the act of navigating culture, not language, that shapes cognition (K. R. Paap et al., 2015; Samuel et al., 2018; Xie et al., 2022). Despite this, culture is often overlooked in cognitive research. A recent review found that only 7% of articles (292/4383) published in cognitive psychology journals from 2016 to 2020 included some element of culture in their study (Gutchess & Rajaram, 2023). Among the subset (7% overall) of articles that did consider some broadly defined aspect of culture, 83% of them focused exclusively on language or bilingualism.

#### Bicultural Identity

The act of navigating bicultural environments and identities is of interest to studies about bilingualism and cognition (Xie et al., 2022). When faced with complex social situations, people with multiple cultural identities can switch between different cultural schemas based on environmental cues (Benet-Martínez et al., 2002; Luna et al., 2008). Tadmor and Tetlock (2006) argue that people who are continuously exposed to a second culture expand the scope of their attention, enabling them to search their environment for cues to help select the appropriate social schema. Furthermore, they argue that when individuals experience strong dissonance between their two cultures in a mixed environment, they are more likely to develop nuanced and complex behavioral schemas that help outline when, why, and to what extent one value is more relevant than another.

In their Bicultural Identity Integration (BII) framework, Benet-Martínez and colleagues propose that there are two components to the integration of cultural identities; blendedness and harmony (Benet-Martínez & Haritatos, 2005; Huynh et al., 2018). The cultural blendedness versus compartmentalization dimension captures the degree to which the individual sees their cultural identities as separate or one and the same. The cultural harmony versus conflict dimension indexes how much dissonance is experienced as a result of conflicting values or perceptions between the two cultural identities.

Ward et al. (2018) distinguish between two additional aspects affecting bicultural identity formation: content vs process. Identity content refers to “what”, the individual elements and structure of identity itself, whereas identity processes refer to the “how”, strategies that bicultural individuals employ to manage multiple cultural identities when responding to shifting identity-related demands. Ward et al. (2018) posit that biculturals dynamically deploy different strategies, hybridizing and alternating to maintain and develop their cultural identities. The hybrid identity style marks a dynamic and flexible process where the multiple cultural identities are combined in novel ways deemed to best represent the identity of an individual. An alternating identity style, on the other hand, denotes a process of upweighting or downweighing a specific cultural frame and switching between the corresponding identities based on situational or context dependent cues.

While the BISS-2 (Huynh et al., 2018) and the MISS (Ward et al., 2018) assess overlapping constructs, other work (Szabó et al., 2020) suggests that correlations between factors from the two scales indicate an important distinction: strategies for maintaining a bicultural identity, as measured by the MISS, align with personal characteristics captured by the BISS-2. Put differently, the MISS measures the strategies through which individuals develop Hybrid and Alternating bicultural identity styles, whereas the BIIS-2 helps explain these styles by assessing characteristic-based components such as personality traits (e.g., neuroticism) and ethnic identity (Ward et al., 2018). These findings suggest the two scales should be used in combination.

The structure and management of bicultural identities have typically been investigated in research focusing on the psychosocial outcomes associated with the acculturation process (Schwartz et al., 2019; Szabó et al., 2020; Ward et al., 2018). Some researchers have begun to investigate their role in cognitive processes associated with bilingualism (Treffers-Daller et al., 2020; Xie & Ng, 2024; Ye et al., 2017; Yim & Clément, 2021).

Treffers-Daller et al. (2020) examined Turkish–English bilinguals who migrated to the U.K. from Cyprus or Turkey. Despite similar linguistic backgrounds, these individuals employed different strategies for managing their cultural identities, shaped by the cultural demands of their respective homelands. They found that individuals from Cyprus who primarily employed an alternating identity style exhibited superior performance on a Flanker task compared to those from Turkey who primarily employed a hybridization identity style. Ye et al. (2017) found that proficient bilinguals outperformed non-proficient bilinguals on the incongruent trials of a flanker task in a mixed cultural context but not in a single culture context. Along similar lines, Xie and Ng (2024) found that people who frequently switched between cultural frames outperformed infrequent switchers on incongruent trials of the Flanker task. Finally, research by Yim and Clément (2021) found that among bicultural individuals, differences in bicultural identification are closely associated with notable variances in their comfort with, preference for, and attitudes towards code-switching, in addition to their perspectives on multiculturalism. These early results collectively underscore the need to investigate these phenomena in new populations in order to understand the relationship between cultural identity and linguistic practices.

### 1.4. Heritage Bilinguals of Latin American Descent in the U.S.

In the context of the United States, descendants of Latin American immigrants represent an ideal population in which to study the intersection of language and culture. At 19.1% of the US population, Hispanic or Latin(o/@/x/é) individuals represent America’s largest and fastest growing racio-ethnic group (Peña et al., 2023; *U.S. Census Bureau QuickFacts: United States*, 2022). Of the estimated 76 million bilinguals in the U.S., 61% of them are English–Spanish bilinguals. Currently, U.S. born Latinés outnumber Latinés born abroad (Funk & Lopez, 2022). Self-rated English proficiency has increased by 19% in the last 30 years, while the use of Spanish in the home has decreased by about 10% (Funk & Lopez, 2022). These shifting demographics give us some insight into the sociolinguistic context of Latinés in the U.S.

Even in locations such as South Florida where Spanish is ubiquitous, studies have found that Spanish is viewed as having lower status when compared to English (Kutlu & Kircher, 2021). In the Southwest United States, the status of both English and Spanish was viewed similarly, but code-switching between the two was held in low regard (Rangel et al., 2015). U.S. Latinés are caught between a rock and a hard place, perceived as inherently low-proficiency English speakers (Flores & Rosa, 2015) while simultaneously judged for not speaking the heritage language (Stransky et al., 2022; Tseng, 2021). While culture has historically been seen as a nuisance variable (Causadias, 2020), language itself emerges from an individual’s interactions with their social environment (Bates, 1976), so any study of language by necessity needs to consider the context in which the language is being used.

### 1.5. Network Approaches to Bilingualism

Titone and Tiv (2022) introduce their Systems Framework of Bilingualism as a way of understanding the interplay between sociolinguistic context, language use, development, and cognition. This framework was inspired by Bronfenbrenner’s (1977) ecological systems theory and situates any study of bilingualism as needing to consider the interplay of the individual, between individuals, and with society at large. This approach acknowledges that bilingual experiences are shaped by a complex web of factors, all of which interact and influence one another. This conceptualization of bilingualism lends itself well to a novel systems approach that conceptualizes behaviors as emerging from the complex and interdependent interactions between psychological, environmental, and/or biological factors, the psychometric network framework (Borsboom et al., 2021).

Psychometric network models offer a significant advantage in exploring complex phenomena by focusing on the specific variance between directly related variables, in contrast to latent variable models that explore the variance shared across measures. In this context, variables like language proficiency and age of onset are linked not through a common latent “bilingualism” construct but shaped through their direct, reciprocal interactions. These models visualize the relationship between variables as networks of nodes (variables) connected by edges (partial correlations), providing a clearer view of the underlying dynamics by isolating the unique interactions among variables.

The partial correlations are closely related to the strengths in multiple regression models; if the independent variable does not predict the dependent variable, then we would not expect to see an edge in the network (Epskamp & Fried, 2018). The biggest draw towards using network models is that they allow you to examine all of the edges that are connected to a node, enabling researchers to concurrently examine all the variables that predict independent variables. In aggregate, this allows researchers to map out linear prediction and multicollinearity through all the variables in the network, enabling the identification of potential mediators in the network (Epskamp & Fried, 2018).

Kałamała et al. (2023) used a psychometric network approach to study Polish–English bilinguals and found that a psychometric network model best captured the complexity of bilingual experiences over traditional latent factor models. They found that self-rated L2 language proficiency, diversity of language use, and language-mixing practices were the measures that were most central (i.e., had the most influence) in the network and captured the most interindividual variability within their sample. In contrast, they found that indices of language onset and language skills likely reflected a specific aspect of bilingualism that was not indicative of the construct as a whole. Importantly, they found that the age of active L2 use mediated all of the edges associated with age of acquisition, which is typically the gold standard in bilingualism research (Surrain & Luk, 2019).

One key limitation of previous studies of bilingualism is the use of methods that overlook interactions among predictor variables. Instead, studies often focus exclusively on the capacity of these variables to explain the variance of the dependent variable. Prior research indicates that many commonly studied variables in bilingualism are highly interrelated (Marian & Hayakawa, 2021; Treffers-Daller, 2018) and form complex systems characterized by reciprocal interactions (Caldwell-Harris & MacWhinney, 2023; Titone & Tiv, 2022). For example, the age at which an individual learns a language influences their language proficiency (Caldwell-Harris & MacWhinney, 2023); their language proficiency can also be dependent on the sustained use of the language (Polinsky & Scontras, 2020), which in turn affects both the frequency and type of code-switching they engage in (Luk & Bialystok, 2013; Schächinger Tenés et al., 2023). Additionally, all of these variables are highly dependent on the norms and values present in the speaker’s sociolinguistic environment (Leeman, 2018; Muysken, 2013; Tseng, 2021). Accurately capturing the intricate network of relationships necessitates modeling approaches capable of accounting for the interdependence among variables.

Compounding this limitation, a recent systematic review of studies on bilingual language use and cognition (Rayo et al., 2024) found that, despite theoretical emphasis on bilingualism as a context-sensitive phenomenon, very few studies directly measured how bilinguals use their languages in everyday life. Instead, most relied on measures such as age of acquisition or language proficiency, with limited consideration of contextual language use, sociocultural factors, or naturalistic code-switching behaviors. As a result, key sources of variability in bilingual experience that are theoretically relevant to observe cognitive outcomes are routinely overlooked.

### 1.6. Present Study

Prior studies looking to determine the existence of a bilingual advantage have focused on predicting the effects of bilingualism on a single cognitive- or identity-related outcome variable. This approach paints an incomplete picture regarding the relationships between bilingual language use, bicultural identity management, and cognition. In the current study, we use an exploratory psychometric network approach to model bilingualism as an emergent system characterized by the dynamic interplay of bilingual language use, bicultural identity management, and cognition. Following Muysken’s (2013) framework for bilingual optimization strategies, we will account for language-specific factors by restricting our population of interest to English–Spanish bilinguals of Latin American descent in the United States. This allows us to investigate the complex relationship between person-specific factors and the sociocultural contexts in which bilinguals and biculturals manage their culture and languages.

Using psychometric network analysis, we explored the relationships among linguistic, cognitive, and sociocultural variables within the context of bilingualism and biculturalism in a population of heritage Spanish–English bilinguals. To achieve this goal, we conducted Bayesian psychometric network analyses to examine how bilingual language practices, strategies for managing bicultural identities, and cognitive abilities interact within a socially infused framework of bilingualism.

We report on the structure and uncertainty of the estimated networks. We then identify the nodes that play a central role in shaping and characterizing the network model of bilingualism using centrality measures while accounting for the uncertainty in these estimates using Bayesian model averaging (BMA) (Hinne et al., 2020). This research builds on our understanding of bilingualism as a dynamic system and identifies future research avenues to shed light on the complex interdependencies between language, identity, and cognition.

## 2. Methods

### 2.1. Participants

Participants were recruited via the online participant recruitment platform Prolific and were paid $12 an hour for their participation. The survey and all questions were presented in English. The median participant spent 49.68 min (MAD = 19.51 min) completing the study and was compensated for two full hours. To be eligible to participate in our study, participants needed to meet the following pre-screening criteria on Prolific: (1) be fluent in both English and Spanish, (2) be bilingual, (3) have no language-related disorders, (4) be born in the United States, (5) be of Hispanic/Latino descent, (6) their first language must be English or Spanish, and (7) they must be at least 18 years old.

A total of n=446 participants met our pre-screening criteria. After removing 32 participants who did not correctly specify “Spanish” as the spoken language for the BSWQ (Rodriguez-Fornells et al., 2012), there were n=414 participants remaining. This criterion was used because participants who left this blank (n=22) or entered in another language as their L2 would have been prompted to respond to incoherent questions, since the text input was used to fill information in their survey items. We then removed participants who did not report a 1st or 2nd language (n=3) or who responded with a language other than English or Spanish (n=1) n=410. Finally, participants who did not meet the inclusion criteria set forth in Hartanto and Yang (2020), i.e., who reported never actively using both of their languages in their daily lives or who have 0% second language exposure, were identified and removed (n=6), since they do not meet the criteria for bilingual interactional contexts. The final sample consisted of n=404 participants.

### 2.2. Measures

#### 2.2.1. Demographics

Participants’ self-reported demographic variables including age, gender, race, ethnicity, family income, educational level, and generational status are included in Table 1. Our sample had an average age of 30.33 years (SD = 9.3 years). In terms of gender identity, 51.2% (n=207) identified as women, 44.1% (n=178) as men, and 4.5% (n=18) as non-binary, and data were missing for 0.2% (n=1) of participants. Regarding generational status, most participants were 2nd generation (80.9%, n=327), followed by 3rd generation (11.9%, n=48), 4th generation and above (5.0%, n=20), 1st generation (1.7%, n=7), and 1.5 generation (0.5%, n=2). All participants in the study reported being born in the United States, so the responses for 1st generation and 1.5 generation may reflect an ongoing disagreement between academics and the general public regarding whether first generation means the first to come to the US or the first to be born here (Contreras, 2024).

Family income varied, with 4.5% (n=18) reporting less than $10,000; 7.2% (n=29) reporting between $10,000 and $19,999; 8.2% (n=33) between $20,000 and $29,999; 12.6% (n=51) between $30,000 and $39,999; 10.6% (n=43) between $40,000 and $49,999; 13.4% (n=54) between $50,000 and $59,999; 7.2% (n=29) between $60,000 and $69,999; 7.9% (n=32) between $70,000 and $79,999; 5.4% (n=22) between $80,000 and $89,999; 6.2% (n=25) between $90,000 and $99,999; 11.6% (n=47) between $100,000 and $149,999; and 5.2% (n=21) reporting more than $150,000.

Racial identity was measured with the option to select more than one category. Aggregating responses across the unique combinations, 66.3% (n=268) of participants identified as White, while 24.5% (n=99) selected “Racial group(s) not mentioned above.” Additionally, 11.6% (n=47) identified as Native American/American Indian/First Nation/Indigenous and 6.2% (n=25) identified as Black. Smaller proportions of participants identified as Asian (2.0%, n=8) and Middle-Eastern/North African (0.7%, n=3), with 0.2% (n=1) identifying as Alaskan Native. In addition, 2.2% (n=9) of responses were recorded as missing. Finally, 11.1% (n=45) of the participants selected multiple racial identities, with their counts contributing to the totals for each individual racial category. For a more in-depth look at the role of mestizaje in shaping racial identity in Latin American history, please see (Chavez-Dueñas et al., 2014).

The overwhelming majority of participants (93.6%, n=378) reported their ethnicity as Latinx/Hispanic. Smaller percentages reported Black, Latinx/Hispanic (3.0%, n=12), White (1.2%, n=5), Indigenous/Native (1.0%, n=4), or an ethnic group not mentioned above (0.5%, n=2). Even fewer identified as African (0.2%, n=1) or Asian (0.2%, n=1), with 0.2% (n=1) of the responses missing. Of the 404 responses provided for specific ethnic identity, the most frequently reported identity was “Mexican” (43.6%, n=176), followed by responses coded as NA (i.e., no specific ethnicity provided) (21.0%, n=85). Next, “Cuban” (5.4%, n=22), “Puerto Rican” (4.7%, n=19), and “Salvadoran” (3.2%, n=13) were the most common specific identities. Responses for “Dominican” (2.7%, n=11) and “Colombian” (2.5%, n=10) were also noted. Less frequently reported identities (e.g., “Mexican–American,” “Guatemalan,” and “Honduran”) ranged from approximately 0.3% to 1.2%. Overall, 9.3% (n=37) of participants reported multiple ethnic identities; for these responses, each component identity was included in the corresponding group count.

#### 2.2.2. Language History and Language Use

Language History Questionnaire (LHQ3) (P. Li et al., 2020). Provides an assessment of the linguistic background of bilinguals in the domains of language use, context and habits of usage, proficiency, dominance, and cultural identity of the languages acquired. The scale consists of 22 items (e.g., “List the languages you have studied or learned, the age at which you started using each language in terms of listening, speaking, reading, and writing”).

In our study, we used the following variables from LHQ-3, Age of Acquisition (AoA)/Age of Contextual use (AoC) for English and Spanish, which were assessed by the items “indicate the age at which you started using each language in terms of listening (AoA), speaking (AoC)”. English and Spanish language proficiency was indexed by the participants’ response to “Rate your current ability in terms of listening, speaking, reading, and writing in each of the languages you have studied or learned (including the native language).” Participants responded using a 7-point Likert scale from 1 (Very Poor) to 7 (Excellent).

Language Use (Use) was calculated via the language use section of the LHQ-3, where participants were asked the percentage of time they spent across four language contexts (family, friends, classmates, and others such as coworkers, roommates, etc.) and the percentage of time they spent using each language in each of those contexts. Spanish language use was calculated by summing the proportion of time spent speaking Spanish in each language context by the proportion of time spent in the corresponding language context.

#### 2.2.3. Switching Between Languages

Language Entropy. The relative diversity of daily language use (Gullifer & Titone, 2020) was calculated using the language use section of the LHQ-3 and the R package languageentropy version 1.0.0 (Gullifer, 2018).

Language Mixing (Mix). The weighted frequency of language mixing (Kałamała et al., 2023) was calculated by multiplying the responses to LHQ-3 questions assessing the frequency of mixing languages in normal conversations for each context on a Likert scale from 0 (None) to 6 (Extreme) by the self-reported proportion of time spent in each of those language contexts.

Bilingual switching questionnaire (BSWQ). The BSWQ (Rodriguez-Fornells et al., 2012) assesses individual differences in language switching within four factors: Language 1 switch (McDonald’s ω = 0.58, SE=0.04, 3 items), Language 2 switch (McDonald’s ω = 0.71, SE=0.03, 3 items), contextual switch (McDonald’s ω = 0.82, SE=0.02, 3 items), and unintended switch (McDonald’s ω = 0.7, SE=0.03, 3 items). The scale consists of 12 items and sample questions include, “I tend to switch languages during a conversation (for example, I switch from L1 to L2 or vice versa)”, and “Without intending to, I sometimes produce the L2 word faster when I am speaking in L1”. Scores for each factor are the average of the responses.

Revised Bilingual Interactional Context Questionnaire (RICQ) (Hartanto & Yang, 2020). Participants are asked to report the prevalence of each interactional context (single-language context, dual-language context, dense code-switching context) as a percentage of time in each of four distinct situations (home, school, work, and others). The percentages of all the interactional contexts in each place must total to 100%. For example, what is the percentage of your language-switching tendency at home? I speak only one language and rarely switch to the other language at home (___%), I speak two (or more languages) when I converse with different speakers at home / I often switch languages but rarely mix languages within an utterance (___%), or I routinely mix two (or more) languages within an utterance to most speakers at home (___%). Participants are also asked to report the percentage of time they spend in each of the four situations (home, school, work, and others).

Indices of single-language, dual-language, and dense code-switching contexts will be calculated to estimate the prevalence of each participant’s type of bilingual interactional context using the following formulas.

$$\mathrm{Single}\text{-}\mathrm{language}\;\mathrm{context}\;\mathrm{index}=\sum_{i=4}^{4}\frac{p_{i}\times sl_{i}}{100}$$

$$\mathrm{Dual}\text{-}\mathrm{language}\;\mathrm{context}\;\mathrm{index}=\sum_{i=4}^{4}\frac{p_{i}\times dl_{i}}{100}$$

$$\mathrm{Dense}\;\mathrm{code}\text{-}\mathrm{switching}\;\mathrm{context}\;\mathrm{index}=\sum_{i=4}^{4}\frac{p_{i}\times dc_{i}}{100}$$

where $p_{i}$ denotes the amount of time spent in each situation; $sl_{i}$ denotes the percentage of a single-language context within a given situation; $dl_{i}$ denotes the percentage of a dual-language context within a given situation; and $dc_{i}$ denotes the percentage of a dense code-switching context within a given situation.

Childhood Revised Bilingual Interactional Context Questionnaire (CRICQ). An adapted version of the RICQ (Hartanto & Yang, 2020) created for this study that asks participants to respond to these questions while thinking of their experiences growing up, before they were 18 years old.

#### 2.2.4. Managing Bicultural Identities

Before being presented with the following measures, participants were asked to respond to the following question “What culture (outside of American) do you identify with? (e.g., Japanese, Mexican, Chinese, Native, Samoan)”. Their typed response was piped into any questions relating to biculturalism, since prior research in our lab has demonstrated that individual responses to these questions are sensitive to the labels used to prime them (e.g., Latinx, Latiné, Hispanic, specific ethnic-group). Both of the Identity integration scales used a 5-point Likert scale (anchored using 1 = strongly disagree to 5 = strongly agree). Scores for each subfactor are the average of the responses.

Bicultural Identity Integration Scale (BIIS-2) (Huynh et al., 2018). This measure is composed of two subscales that index cognitive (blendedness vs. compartmentalization) and affective (harmony vs. conflict) influences on identity formation across the experiences of bicultural individuals. The blendedness (McDonald’s ω = 0.84, SE=0.01) subscale measures the extent to which an individual considers their cultural identities to be distinct and separable, e.g., I relate better to a combined ______ American culture than to ______ or American culture alone. The harmony subscale (McDonald’s ω = 0.89, SE=0.01) contains items assessing the subjective level of conflict experienced as a result of managing distinct cultures, e.g., I rarely feel conflicted about being bicultural.

Multicultural Identity Styles Scale (MISS) (Ward et al., 2018). The MISS assesses the strategies that multicultural individuals employ to manage their multiple identities. The two subscales reflect the utilization of distinct identity styles where the hybrid identity style reflects the blending of both cultural identities in the creation of a new cultural mélange and the Alternating identity style reflects an identity strategy where different components of their cultural identities are emphasized depending on the environmental context. The hybrid (McDonald’s ω = 0.86, SE=0.01) and alternating (McDonald’s ω = 0.84, SE=0.01) subscales each contain seven items. For example, Hybrid: For me, being ______ and being American are intermingled; Alternating; I alternate between being ______ and American, depending on the circumstances.

#### 2.2.5. Attentional Control

Participants completed the jsPsych (de Leeuw et al., 2023) version of the three “Squared” attentional control tasks (Stroop, Flanker, and Simon) introduced by Burgoyne et al. (2023) and adapted for jsPsych by Liceralde and Burgoyne (2023). The tasks incorporate an additional level of conflict by challenging participants to resolve incongruencies present in both the display and response options. For example, in the Stroop Squared task, a central target word, either “RED” or “BLUE,” appears in red or blue color. Participants must first respond to the color of the word (as in the classic Stroop task), then select one of two response options below based on semantic content, ignoring their color. The additional challenge arises from simultaneously managing conflicting color and semantic cues, e.g., attending to the display color of the central stimulus and the meaning of the response options, while disregarding the stimulus’s meaning and the responses’ display color. We followed the task order established in Burgoyne et al. (2023) where all participants completed the Squared tasks in the same order, (1) Stroop, (2) Flanker, and (3) Simon, but the trial order is randomized. In the three “Squared” tasks, participants begin with an example item (incongruent) and instructions on how to complete the task. Participants complete a 30 s practice phase followed by a chance to review the instructions and then continue on to the 90 s test phase. Participants receive item-level feedback on all trials. Reliability was calculated using split-half internal consistency estimates using the Spearman–Brown prophecy formula.

Stroop Squared. Participants must match the display color of the target stimulus with the semantic meaning of one of the two response options. Performance on the task was measured by the number of correct responses minus the number of incorrect responses during the 90 s test phase. (M=26.27, SD=16.25, split-half reliability = 0.91).

Flanker Squared. Participants must match the direction of the flanking arrows of the target stimulus with the direction of the central arrow of one of two response options. Performance on the task will be measured by the number of correct responses minus the number of incorrect responses during the 90 s test phase. (M=20.98, SD=15.87, split-half reliability = 0.90).

Simon Squared. Participants must match the direction that a target stimulus arrow is pointing with the semantic meaning of one of two response options. Performance on the task will be measured by the number of correct responses minus the number of incorrect responses during the 90 s test phase. (M=50.06, SD=17.69, split half reliability = 0.91).

Fluid Intelligence. Raven’s Advanced Progressive Matrices (RAPM). Participants were presented with Bors and Stokes (1998) a short form of RAPM set II (Items 3, 10, 12, 15, 16, 18, 21, 22, 28, 30, 31, and 34). This task presents participants with a 3 × 3 matrix of patterns with the bottom-right pattern missing. Participants select which of the available 8 options completes the series. The task was administered in its power form (untimed) as a recent meta-analysis found that a speeded (timed) administration reduced the test’s loading on fluid intelligence (Tatel et al., 2022). The critical measure is the total sum of correct responses (0–12). (McDonald’s ω = 0.69, split half reliability α = 0.68).

### 2.3. Procedures

The data was collected on the participant recruitment site Prolific (Prolific, 2025). Participants who met our inclusion criteria on prolific were eligible to sign up to participate in our study. Once enrolled, participants were directed to Qualtrics (Qualtrics, 2024) to complete the following questionnaires. After confirming that they met our inclusion criteria, participants were asked to fill out demographic questions: the Language History Questionnaire (LHQ-3) (P. Li et al., 2020), the revised version of the Code-Switching and Interactional Contexts Questionnaire (RICQ) (Hartanto & Yang, 2020), a modified version of the RICQ created for our study that asks participants about the interactional contexts they experienced during their childhood (Childhood RICQ), the Multicultural Identity Style Scale (MISS; (Ward et al., 2018)), the Bicultural Identity Integration Scale (BIIS-2; (Huynh et al., 2018)), the Bilingual Switching Questionnaire (BSWQ; (Rodriguez-Fornells et al., 2012)), and Raven’s Advanced Progressive Matrices (RAPM; (Bors & Stokes, 1998)). After completing all of the survey questionnaires and the RAPM, participants were redirected to an outside program (E-Prime GO (*E-Prime Go*, 2020) or Pavlovia (*Pavlovia*, 2018)) for behavioral data collection to complete the three squared attentional control tasks (Burgoyne et al., 2023).

Attentional Control Behavioral Data. The behavioral data collection for the three attentional control tasks occurred in two waves, Wave 1 (235/404) and Wave 2 (169/404 in final sample). The initial wave of data collection was conducted using E-Prime GO (*E-Prime Go*, 2020). This change in data collection methodology was enacted to address issues that participants were having with completing the attentional control tasks. Over the course of our study, we discovered that a subset of our participants were experiencing some sort of computer-related error that prevented them from running the tasks, successfully completing the tasks, or even uploading the resulting task score CSVs (as a failsafe). Of the 235 participants who completed the E-Prime GO behavioral tasks, 158 (67.2%), 152 (64.7%), and 154 (65.5%) participants successfully completed the Stroop, Flanker, and Simon tasks, respectively. For the remainder of the study, data was collected using jsPsych (de Leeuw et al., 2023) on Pavlovia.org (*Pavlovia*, 2018), which presents cognitive behavioral tasks via browser using JavaScript. Of the 169 Participants who completed the task on Pavlovia, 155 (91.7%) successfully completed the tasks. We conducted a series of Bayesian Independent Samples T-Tests to examine whether there were differences across the two waves and only found differences in the Simon task where we found extreme evidence to support there being differences between the two waves (${BF}_{10}=571.04$). As a result, the scores on the Simon task will be excluded from our subsequent analyses.

### 2.4. Variable Selection

In accordance with the reporting standards for psychological network analyses in cross-sectional data (Burger et al., 2023), we report on the following variable selection procedures we conducted: missing data, checking for violations of assumptions, subsequent data transformations, and node redundancy.

We first analyzed our missing data to determine if the data were missing at random (MAR), since network estimation procedures often employ built-in mechanisms for handling missing data such as pairwise estimation, imputation, or listwise deletion (Epskamp et al., 2018; Huth et al., 2023). Our data met the criteria for MAR, meaning that missingness is random after accounting for observed data (Enders, 2023; van Buuren, 2018), which in our case is the source of the data, E-Prime GO vs Pavlovia.

Missing data were imputed using Fully Conditional Specification (FCS) (Enders, 2023; van Buuren, 2018). The imputation was conducted using the multiple imputation by chained equations (MICE) algorithm for the imputation of multivariate missing data (van Buuren & Groothuis-Oudshoorn, 2011). The MICE algorithm imputes variables one at a time using a series of univariate regression models. The algorithm starts with a random draw from the observed data and imputes the incomplete data in a variable by variable fashion. We followed the guidelines for imputation from van Buuren (2018) and imputed the data over 40 iterations using the MICE package (van Buuren et al., 2024). The variables were then centered and scaled prior to network estimation.

The overall level of node redundancy was estimated via the network measure, weighted topological overlap (wTO), which quantifies how much similarity is shared between the partial correlations for nodes across the network in order to detect violations of local independence (Christensen et al., 2023). Since network models do not traditionally account for latent variables, wTO can be used to identify nodes that have too many shared edges (measured via edge weights, signs, and quantity). If nodes have too much topological overlap (wTO > 0.25), then model estimation can be impacted, as well as the estimation of model parameters, inaccuracies in estimating the internal structure, and any subsequent estimation of other network properties such as network centrality measures. Weighted topological overlap (wTO) was calculated using Unique Variable Analysis (Christensen et al., 2023).

To select which variables to keep and which to remove from redundant pairs, the default heuristic procedure in the uva function was used (Christensen et al., 2023). The item with the lowest maximum wTO was retained and the other items were removed from the network estimation procedures. This method was chosen because the variable with the lowest wTO is the variable that possesses the most unique information. The unique variable analysis identified several pairs of nodes with high weighted topological overlap (wTO), indicating that these play similar roles in their connectivity across the network, i.e., they are highly redundant. The threshold used was a wTO greater than 0.25 and we retained the node with the lowest maximum wTO, retaining the node that accounted for more unique variance across the network. AoAeng with AoCeng (wTO = 0.709), Biis_blendedness with MISS_Hybrid (wTO = 0.536), and AoAspa with AoCspa (wTO = 0.35), as well as pairs like DCi with CDCi (wTO = 0.324), DLi with CDLi (wTO = 0.317), and entropy with usesspa (wTO = 0.274).

Skip Structures: Both bilingual interactional context questionnaires (RICQ and CRICQ) contain skip structures. For example, if a participant recorded spending 0 time with classmates, we did not ask them to break down the percentage of time spent in each interactional context with their classmates, so those questions would contain missing values. Subsequently, we then imputed 0s for these missing values. According to the guidelines for network estimation provided by Burger et al. (2023), skip structures can introduce dependencies in the data. However, according to Borsboom et al. (2017), although this practice can affect the estimation of network models, it does not invalidate the results as long as the skipped items meet the criteria for Guttman scale properties (e.g., if one does not spend time with classmates, then one cannot break down the kind of language environment that is present in a non-existent context). Since our skip-structure data meet these criteria, we retained the items in our analyses.

### 2.5. Network Visualization

Networks were visualized using the Fruchterman–Reingold algorithm (Fruchterman & Reingold, 1991) (“spring” setting) in the qgraph package. This algorithm is a force-directed graphing methodology that determines node placement by simulating nodes as repelling each other and edges as holding nodes together.

### 2.6. Network Estimation

A Gaussian copula graphical model (GCGM) was estimated using a Bayesian framework (Huth et al., 2023). This approach allows us to use graphical models in order to estimate the conditional dependencies and partial correlations between our variables while the use of Bayesian methods allows us to quantify the level of uncertainty in the estimates. In a graphical model, the conditional dependencies between variables are represented in a graph. Variables in the estimated network are defined as nodes and the partial correlations between the variables after accounting for the influence of covariates are represented as edges (Borsboom et al., 2021; Huth et al., 2024). A Gaussian copula graphical model (GCGM) was used over a Gaussian graphical model (GGM) because it does not require variables to be multivariate normal and can model non-Gaussian multivariate data by representing the relationship using latent variables (A. Mohammadi et al., 2017).

The network is estimated from the nodes that correspond to bilingual language use, bicultural identity management, and cognition variables, and a set of edges between nodes that represent the partial correlations between variables. In our network, if two nodes share an edge, it means that they are conditionally dependent after controlling for the effect of all the other nodes in the network (i.e., a partial correlation). Conversely, if an edge is absent, it means that these variables are conditionally independent given the remaining variables (A. Mohammadi et al., 2017). The estimated network is weighted, meaning that each edge has a value that corresponds to the strength of the partial correlation. Edges can be positive, representing a positive association between nodes, or they can be negative, representing a negative association between nodes (Borsboom et al., 2021).

#### 2.6.1. Bayesian Network Estimation

We implemented a Bayesian estimation approach to address uncertainty in our network models. This approach provides two key advantages; it quantifies the uncertainty associated with a network structure (i.e., probability of including/excluding specific edges) and the parameter estimates (i.e., how certain are the edge weights and centrality estimates) (Huth et al., 2024). Importantly, Bayesian estimation allows us to quantify the evidence for the absence of an edge, not just its presence, enabling us to determine which edges are not likely to exist in the network. BDgraph was selected because it uses Bayesian model averaging (BMA) in estimating the partial correlation network, and reports posterior inclusion probabilities, and the structures visited by the MCMC estimation algorithm (R. Mohammadi & Wit, 2019).

When estimating network structure, it is unlikely that a single network structure dominates the posterior model distribution. A likelier alternative is that several models who might differ by a few edges are similarly probable (Hinne et al., 2020). Bayesian model averaging (BMA) (Hinne et al., 2020) treats each possible graph as a statistical model and aggregates the posterior probability of inclusion across all possible structures that contain the edge ($H_{1}$) vs all other possible model structures where the edge was not present ($H_{0}$) instead of just relying on the most likely model. By examining the posterior distribution, we can make the claim that “two nodes are conditionally dependent with a probability of X” (Vogels et al., 2024), e.g., the edge between Spanish language proficiency and the tendency to switch to English was present in 97% of the simulated network structures.

Bayes Factors (BF) for each edge were calculated from the model averaged posterior inclusion probability. The Bayes Factor provides the ratio of probability observed from the data in support of a particular hypothesis—in our case, the evidence that there is ($H_{1}$) or there isn’t ($H_{0}$) an edge (a partial correlation) between these two nodes after accounting for all of the other nodes in the network. For graphically representing our network’s edges, we follow the recommendations of prior researchers (Barbieri et al., 2021; Huth et al., 2023) and use the median probability model where all edges with a posterior inclusion probability greater than 0.5 (BF>1) are included in the network. We chose to represent the strength of evidence for edge inclusion in our estimated network by depicting edges with a Bayes Factor greater than or equal to 10 (represented by a solid edge), which represents a ratio of ten times more support for the alternative ($H_{1}$) than the null ($H_{0}$). This is a common threshold for a strong level of support for the alternative hypothesis, whereas dashed edges in the graph represent edges with a Bayes Factor ranging from 1 to less than 10, which represent anywhere from an anecdotal (BF ranging from 1 to 2.99) to a moderate (BF ranging from 3 to 9.99) level of evidence supporting the alternative hypothesis (Lee & Wagenmakers, 2014). The sampler was set to run for 100,000 iterations and the burnin for 50,000, as recommended by prior literature (Huth et al., 2024).

Another benefit of Bayesian methods is that you can incorporate previously held beliefs about the expected relationship between variables; these beliefs are captured in a distribution called the prior (Kruschke & Liddell, 2018). In BDgraph, the prior takes the form of a value between 0 and 1 and represents the probability of edge inclusion, with 0 representing exclusion and 1 representing inclusion (Huth et al., 2024; A. Mohammadi & Wit, 2015). In our study, we used the default priors provided by the BDgraph and BGGM package, an uninformative prior of 0.5 that deems every edge is equally likely to be included or excluded (R. Mohammadi & Wit, 2019; D. R. Williams & Mulder, 2020). The prior distribution of the precision matrix was set using the BDgraph default of 3 for the degrees of freedom for G-Wishart distribution. The MCMC algorithm used was the birthdeath MCMC (bdmcmc) algorithm detailed in A. Mohammadi and Wit (2015), and an empty starting point was used for constructing the graph.

#### 2.6.2. Accuracy and Stability of Edge Estimates

Accuracy of the overall estimated network was investigated via three metrics for structural uncertainty, the posterior structure probabilities of all simulated structures and the posterior probability of graph complexity, i.e., the number of edges (Huth et al., 2024). Finally, the resulting graph structure for the network shows all edges that have some evidence for inclusion (BF>1). The uncertainty of edge estimates was investigated through the posterior estimates of the edge weights and their 95% highest density intervals. The 95% HDI is the shortest interval that contains 95% of the posterior mass (Huth et al., 2023).

Based on their Bayes Factor, edges will be classified into one of three categories, included, excluded or inconclusive. Included edges will have a Bayes Factor greater than 10 representing a ratio of ten times more support for the alternative ($H_{1}$) than the null ($H_{0}$). Excluded edges will have a Bayes Factor less than 1/10, representing a ratio of ten times more support for the null ($H_{0}$) than the alternative ($H_{1}$). Edges with a Bayes Factor that falls between these thresholds will be categorized as inconclusive. By summing the number of included, excluded, and inconclusive edges in the network, you can determine the total possible number of edges. The higher the proportion of edges that are classified as included or excluded, the more stable the network, i.e., if a network has a higher proportion of inconclusive edges, this would mean that it is highly uncertain whether edges are present or not.

### 2.7. Statistical Packages

The analyses were conducted using R version 4.4.2 (R Core Team, 2021). The tidyverse package version 2.0.0 (Wickham & RStudio, 2023) was used to facilitate data handling and coding for all analyses conducted in R. The *describe* function of the psych package version 2.4.12 (Revelle, 2024) was used to calculate descriptive statistics. The *md.pattern* function of the mice package version 3.17.0 was used to visualize and inspect missing data. For network estimation, we used the *easybgm* function in the easybgm package version 0.1.2 (Huth et al., 2024) as a wrapper for the BDgraph network estimation method of the GCGM (R. Mohammadi & Wit, 2019). Network visualization was conducted using the qgraph package version 1.9.8 (Epskamp et al., 2012). Node redundancy was calculated using the *uva* function of the EGAnet package version 2.1.0 (Golino et al., 2024). The analyses presented in the results were conducted on our imputed sample of n=404.

### 2.8. Network Measures

To quantify nodes that are influential in our estimated network, we investigated the network centrality measures, strength and bridge strength in addition to nodewise predictability.

Strength Centrality. The level of uncertainty of the centrality estimates is represented by the highest density interval for each edge. Strength centrality is calculated as the sum of the absolute edge weights connected to a given node. The level of uncertainty of the centrality estimates is represented by the 95% highest density interval for each edge. These are estimated from the posterior parameter distribution with narrower highest density intervals representing more stable parameters.

Bridge Strength Centrality. In order to identify the nodes that have the strongest influence between our three domains, we used a centrality measure that accounts for community membership, bridge centrality (P. J. Jones et al., 2021). Similar to strength centrality, bridge strength centrality is calculated by summing a node’s absolute edge weights; however, in this case, only the edges that connect to other communities are included. For example, the edge weights between two language variables would not be counted, whereas the edge weights between a language variable and a cognitive variable or a language variable and an identity variable would. This network centrality measure was calculated using the bridge function from the networktools package (P. Jones, 2025).

Predictability. Node predictability is a measure of how well a node can be predicted by all the other nodes in the network (Haslbeck & Waldorp, 2018). For our variables, Predictability is calculated using Bayesian R^2^, defined as the variance of the predicted values divided by the variance of predicted values plus the expected variance of the errors (Gelman et al., 2019). This network measure was calculated using the predictability function from the BGGM package (D. R. Williams & Mulder, 2020).

Both Strength centrality and Predictability were estimated using Bayesian model averaging (BMA) in order to account for uncertainty across the estimated network structures (Hinne et al., 2020). Under BMA, these quantities are not computed from a single “best” network; instead, they are calculated within each sampled model and then averaged across the posterior distribution of all candidate models, with each contribution weighted by the corresponding posterior model probability. Accordingly, these estimates incorporate uncertainty over the full model space. The uncertainty of the resulting parameter estimates is summarized using 95% highest density intervals from the posterior parameter distribution, with narrower intervals representing more stable estimates.

Prior Robustness. Since tests and estimates in Bayesian approaches are influenced by the priors that are specified, we assess the sensitivity of our estimates via a prior robustness check. We reran the analysis for our Spanish–English bilinguals five times and changed the prior edge inclusion probability for each run. Edge priors were set to 0.1, 0.3, 0.5, 0.7, and 0.9, respectively. We discuss prior robustness in terms of how the edge categorization changes across different prior inclusion probabilities.

## 3. Results

Prior to the psychometric network analysis, the variables were centered and scaled in order to ensure a common measurement scale.

### 3.1. Identifying Redundant Variables

To ensure that our network reflected conditional dependencies between variables and not just similarity, we used the network measure, weighted topological overlap (wTO), to identify variables (nodes) that shared too many similar edges within the network (Christensen et al., 2023). The unique variable analysis (UVA) identified certain nodes as being redundant in our current network (wTO>0.25, indicating shared edge patterns), e.g., AoAeng with AoCeng (wTO=0.709), Biis_blendedness with MISS_Hybrid (wTO=0.536), and AoAspa with AoCspa (wTO=0.35), as well as pairs like DCi with CDCi (wTO=0.324), DLi with CDLi (wTO=0.317), and entropy with usesspa (wTO=0.274). These variables were identified as redundant because they shared too many similarly weighted/signed edges with another node in our network. It is important to note that these nodes are deemed redundant given our specific population and given the exact set of measures that were included in the UVA. For example, our results should not be interpreted to broadly mean that the age of speaking the language is a more informative measure than the age of hearing the language.

The two nodes that shared the highest edge overlap (wTO=0.709) were the English language history indices, Age of acquisition (AoA) and Age of contextual use (AoC). The majority of heritage bilinguals of Latin American descent growing up in the United States learn Spanish as their first language and then learn English at a subsequent point, often when they begin schooling (Surrain, 2021). Participants in our sample were exposed to Spanish and began to speak Spanish at an early age (AoA, M=1.231 SD=1.656; AoC, M=2.604 SD=2.884). They were also exposed to English and began to speak English at an early age (AoA, M=2.103 SD=2.063; AoC, M=2.897 SD=2.334). As such the exposure to English and their subsequent use of the language are closely linked. This overlap would not be expected to replicate if we expanded our sample to include people who immigrated to the U.S. or if our participants were from a social context where individuals maintained dominance in their L1. However, the stronger overlap between the English measures (wTO=0.709) compared to Spanish measures (wTO=0.35) suggests greater variability in how and when participants began actively using their heritage language, despite similar exposure timelines.

Since participants in our sample tended to use Spanish in environments where the use of English was also common, either due to code-switching or speaking multiple languages in the same location, language entropy was identified as redundant compared to our weighted measure of Spanish language use. Another factor that likely contributed to this is the prevalence of other indices of code-switching, which greatly reduced the unique contribution of language entropy. Similarly, the measures of participants’ current interactional contexts (DLi/DCi) were likely identified as redundant compared to their childhood counterparts (CDLi/CDCi), not because they were uninformative, but because our network already included multiple measures capturing current language use. The childhood context measures, however, contributed unique developmental information not captured by other variables in our network.

Finally, the substantial overlap between the BIIS-2 (Huynh et al., 2018) measure of blendedness vs. compartmentalization and the measure of hybridizing identity style from the MISS (Ward et al., 2018) (wTO=0.536) indicates a similar set of relationships between cultural identity and language use for heritage Spanish–English bilinguals, regardless of whether we focus on the structure of bicultural identities or the strategies used to manage them.

After addressing measurement redundancy, our network includes variables that each contribute unique information about bilingual/bicultural experiences and cognitive outcomes. This allows us to use psychometric network models to address our research question: How do bilingual language practices, strategies for managing bicultural identity, and cognitive abilities interact within a socially infused framework of bilingualism?

### 3.2. Bayesian Psychometric Networks

The aim of our research was to investigate the relationship between bilingual language use, bicultural identity management, and cognition. Nodes assessing language include profspa, AoCspa, and AoCeng, which assess Spanish language proficiency and the ages at which participants first spoke Spanish and English. Other language nodes include the weighted frequency of language mixing (Mix) or weighted proportion of Spanish use (usespa) across various social contexts, such as with family, friends, classmates, and others; tendencies to switch into English (BSWQ_L2S) or Spanish (BSWQ_L1S); and the prevalence of both contextual (BSWQ_CS) and unintended switches (BSWQ_US). Two nodes indexed their childhood language environments, a dual-language context where they alternated between languages according to who they were talking to and/or without switching within sentences (CDLi), and a dense code-switching context where they often switched languages mid utterance (CDCi). Bicultural Identity measures included the blending of bicultural identities (MISS_Hybrid), context-based cultural identity (MISS_Alternating), and the level of harmony/conflict arising from managing distinct cultures (Biis_Harmony). Cognitive performance nodes measured fluid reasoning (RAPM) and attentional control squared tasks (Stroop and Flanker).

#### 3.2.1. Network Visualization

**Figure 1:**
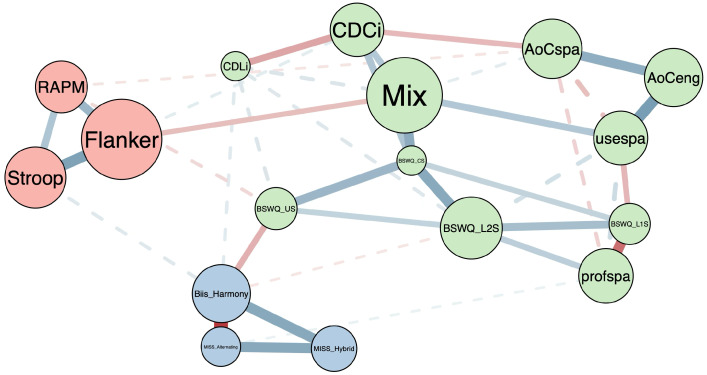
Network visualization of the language, cultural identity, and cognition variables. An edge between nodes represents a conditional dependence with a probability of at least 50% (BF>1), a dashed edge represents edges with a BF greater than 1 but less than 10, and a solid edge represents edges with a BF greater than 10. A blue edge denotes a positive partial correlation, and a red edge is negative. Edge thickness and saturation represent the strength of the association; the stronger the association, the thicker the edge. The size of the node depicts the Bayesian model averaged strength centrality for a given node. Color denotes the broad category that the underlying measure reflects. Nodes assessing language history (green) include profspa, AoCspa, and AoCeng, which assess Spanish language proficiency and the ages at which participants first spoke Spanish and English. Nodes that assess language usage evaluate aspects of bilingual interaction, such as the weighted frequency of language mixing (Mix) or weighted proportion of Spanish use (usespa) across various social contexts such as with family, friends, classmates, and others; tendencies to switch into English (BSWQ_L2S) or Spanish (BSWQ_L1S); and the prevalence of both contextual (BSWQ_CS) and unintended switches (BSWQ_US). They also evaluate the prevalence of the following language contexts during childhood—a dual-language context where they alternated between languages according to who they were talking to and/or without switching within sentences (CDLi), and a dense code-switching context where they often switched languages mid utterance (CDCi). Bicultural Identity measures (blue) included the blending of bicultural identities (MISS_Hybrid), context-based cultural identity (MISS_Alternating), and the level of harmony/conflict arising from managing distinct cultures (Biis_Harmony). Cognitive performance nodes (red) measured fluid reasoning (RAPM) and attentional control squared tasks (Stroop and Flanker).

The network is visualized in Figure 1. Here, the edges represent the partial correlation network between 17 nodes. The visualized network is the median probability network where edges (39/136) with a posterior inclusion probability greater than 0.5 were included in the graph Barbieri et al. (2021). The weight of the smallest edge with an inclusion probability greater than 0.5 was 0.056 (profspa-MISS_Alternating), and the smallest edge with a Bayes factor greater than 10 was −0.144 (BSWQ_US-RAPM) and the largest was −0.441 (MISS_Alternating-Biis_Harmony).

#### 3.2.2. Network Density and Average Absolute Edge Weights

**Figure 2:**
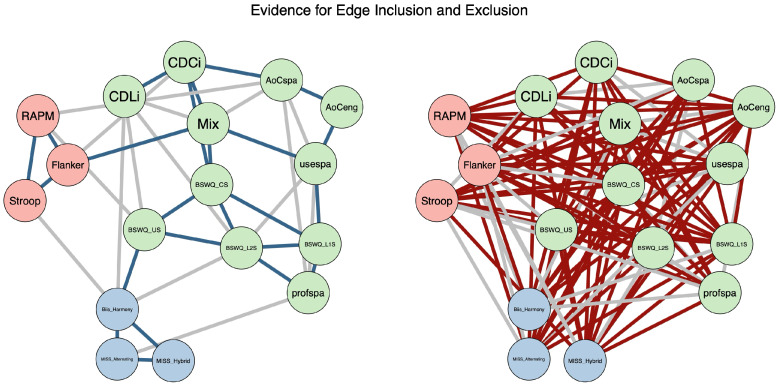
The edge evidence plot depicts the level of support for inclusion or exclusion of an edge based on the Bayes factor. Red edges indicate evidence for edge absence (i.e., conditional independence, ${BF}_{10}<0.10$), blue edges indicate evidence for edge presence (i.e., conditional dependence, ${BF}_{10}>10$), and grey edges indicate the absence of evidence (i.e., a BF between 1/10 and 10).

Table 2 contains the estimated weighted adjacency matrix for our network. The estimated network had 24 edges with sufficient evidence for inclusion (${BF}_{10}>10$), 71 edges with sufficient evidence for exclusion (${BF}_{10}<1/10$), and 41 edges with inconclusive evidence (i.e., a BF between 1/10 and 10). See Figure 2 for a visualization of the edge evidence plot. The network of bilinguals had a density of 0.176 (24/136 edges), a sparsity of 0.824 (112/136 edges), and a mean absolute edge weight of 0.057. The percentage of categorized edges in a network provides an estimate of the network’s stability; the higher the percentage of inconclusive edges, the higher the uncertainty regarding the structure of the network (Huth et al., 2024). In our network, 69.85% of edges (95/136) had strong evidence for inclusion/exclusion, suggesting that the estimated network is suitable for inferential conclusions (Huth et al., 2024).

#### 3.2.3. Structure Uncertainty

**Figure 3:**
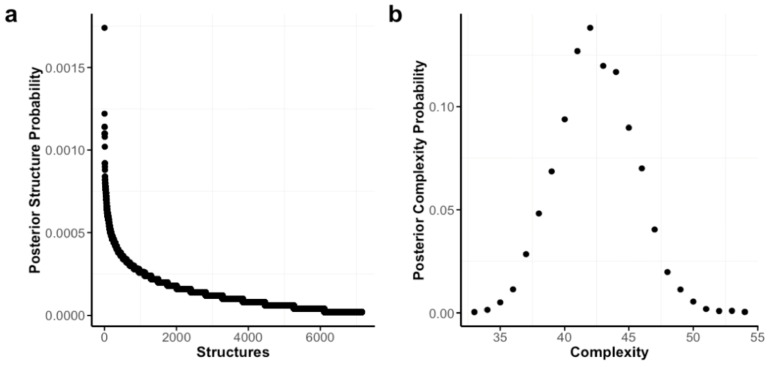
Structure Uncertainty Plots. The posterior structure plot (**a**) shows the posterior probabilities of the visited structures, sorted in the order of probability from most to least. The posterior complexity graph (**b**) shows the posterior probability distribution aggregated over the number of edges.

There are 2^136^ or 8.71 × 10^40^ possible network models for our network (2^k(k−1)/2^), and a priori, we assigned equal probability to all of them. Results are shown in Figure 3. The MCMC algorithm visited 7156 distinct network structures. Even though six network structures show a higher posterior probability than the rest, no single structure dominates the posterior probability. This means that there is a high degree of uncertainty regarding the most probable underlying structure, and there are several plausible structures that could have generated this data. Table 3 shows the probability of the top 10 most likely models. The differences between the most likely models are small. For instance, the top model is only 1.43 times more likely to have generated the data than the 2nd best model, and only 1.89 times more likely than the 10th best model. Although there is considerable uncertainty about which network structure is most likely, we can still examine the overall complexity of the possible structures. Part b of Figure 3 shows that the posterior probability for the number of included edges peaks around 42, and that more than 75% of the posterior probability falls between 40 and 46 edges. So, even though the exact structure is uncertain, we can be relatively confident about its overall complexity.

#### 3.2.4. Parameter Estimation and Precision

**Figure 4:**
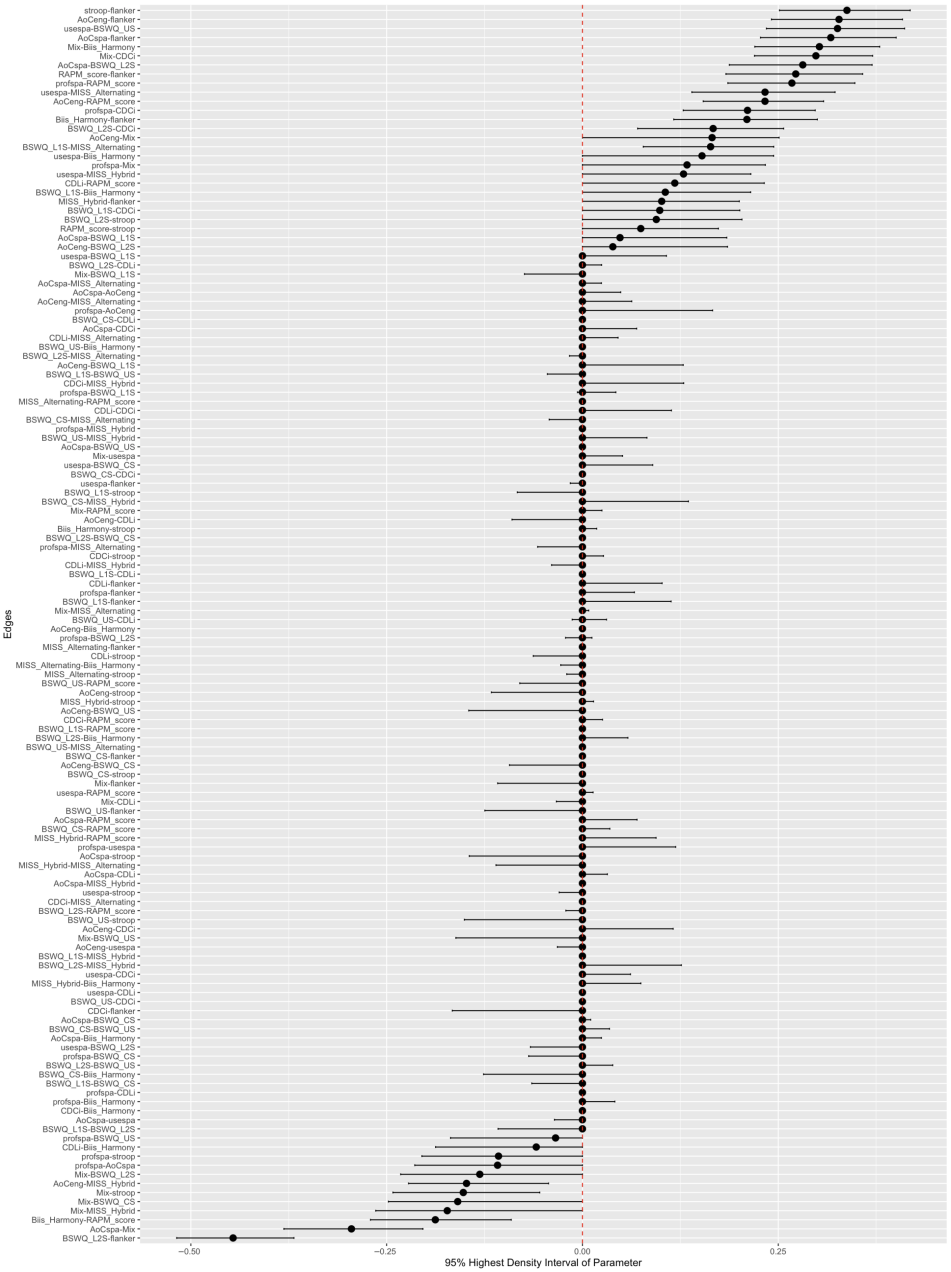
Parameter highest density plot. The plot shows the posterior estimates of the edge weights and their 95% highest density interval.

In order to investigate the uncertainty associated with our edge weights, we visualized the 95% highest density interval of the edge weight posteriors in Figure 4. The x-axis indicates the strength of the model averaged edge weight. The y-axis represents the edge between nodes i and j. The farther the posterior estimates are from zero, the stronger the estimated edge weight. The larger the width of the highest density interval, the more uncertainty there is surrounding the edge weight.

#### 3.2.5. Network Measures

The raw computed values for strength centrality, bridge strength centrality, and predictability in the network are reported in Table 4.

**Figure 5:**
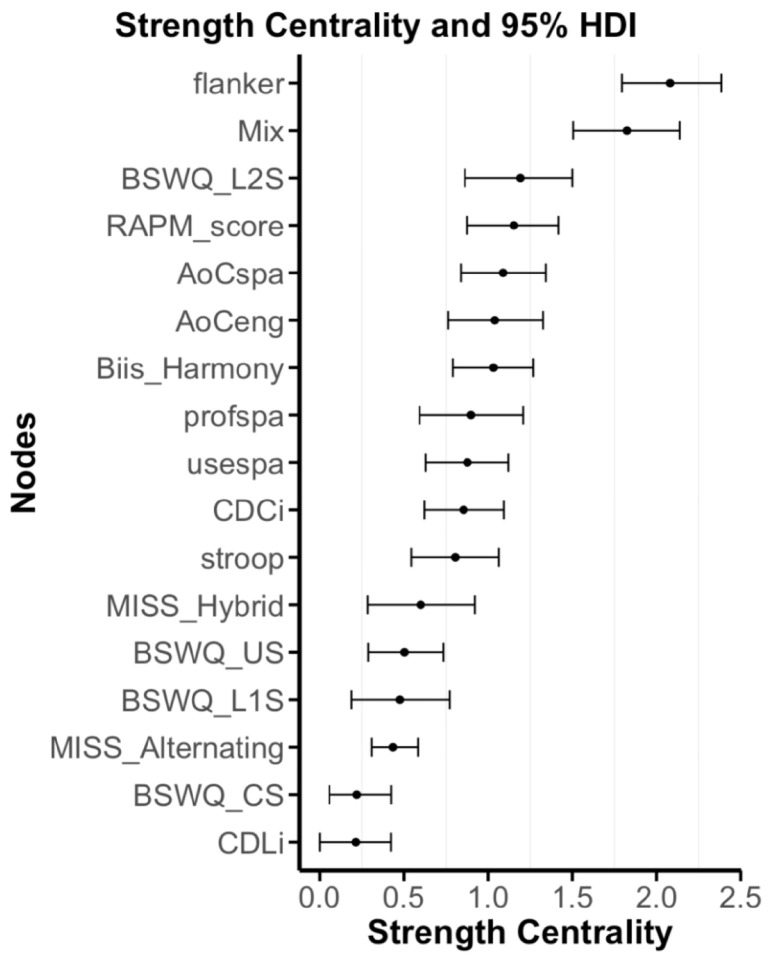
Strength centrality. Result for Bayesian model averaged strength centrality and the 95% highest density interval.

Strength Centrality. Figure 5 presents the results of the strength centrality analysis and the corresponding 95% highest density interval across models. Strength centrality reflects the degree of influence or interconnectedness that a node has on its surrounding nodes in the network. The nodes with the highest strength centrality were Flanker (M=2.08), Mix (M=1.82), BSWQ_L2S (M=1.19), RAPM (M=1.15), and AoCspa (M=1.09). Due to our lower sample size, the 95% highest density intervals trend wider reflecting higher degrees of uncertainty, and thus we need to interpret them with caution.

**Figure 6:**
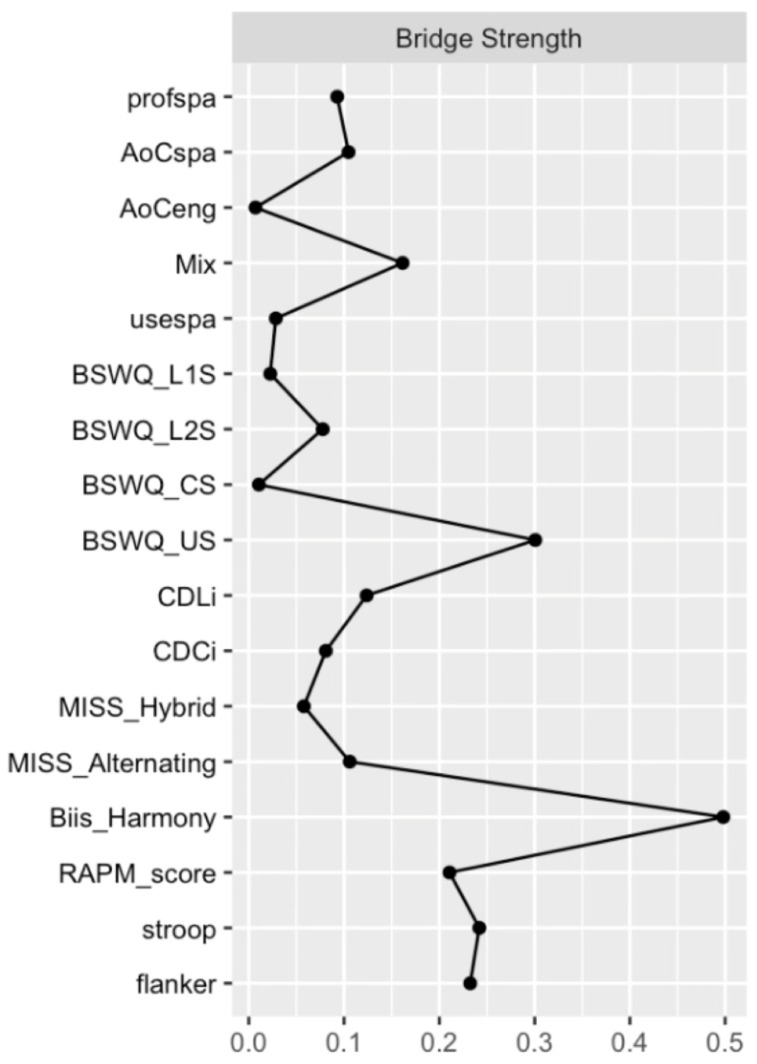
Bridge strength centrality. Result for bridge strength centrality, nodes that have the greatest influence on nodes outside their domain.

Bridge Strength Centrality.The bridge strength centrality analysis identified nodes with the greatest degree of influence outside of their own respective language, identity, and cognition community. Figure 6 depicts a plot of the nodes with their corresponding bridge strength centrality. The nodes with the highest bridge strength centrality were Biis_Harmony (M=0.50), BSWQ_US (M=0.30), Flanker (M=0.24), Stroop (M=0.23), RAPM (M=0.21), and Mix (M=0.16).

**Figure 7:**
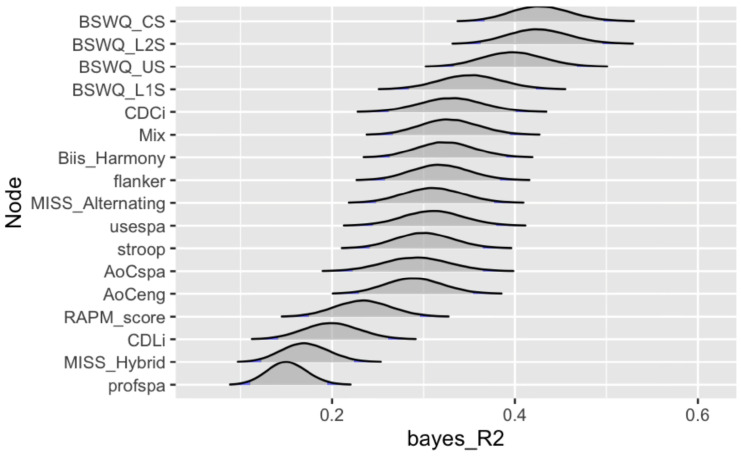
The Bayesian R^2^ predictability measures how well a node can be predicted by all the other nodes in the network. The figure depicts a ridgeline plot of the distribution of R^2^ score per node.

Predictability. The Bayesian R^2^ predictability analysis identified contextual switching (BSWQ_CS) as having the highest R^2^ score (42.9%) in the network, while Spanish language proficiency was the lowest R^2^ score (15.1%) in the network. The Average R^2^ score is 30.4%. Figure 7 depicts a ridgeline plot of the distribution of R^2^ score per node.

#### 3.2.6. Sensitivity Analysis

**Figure 8:**
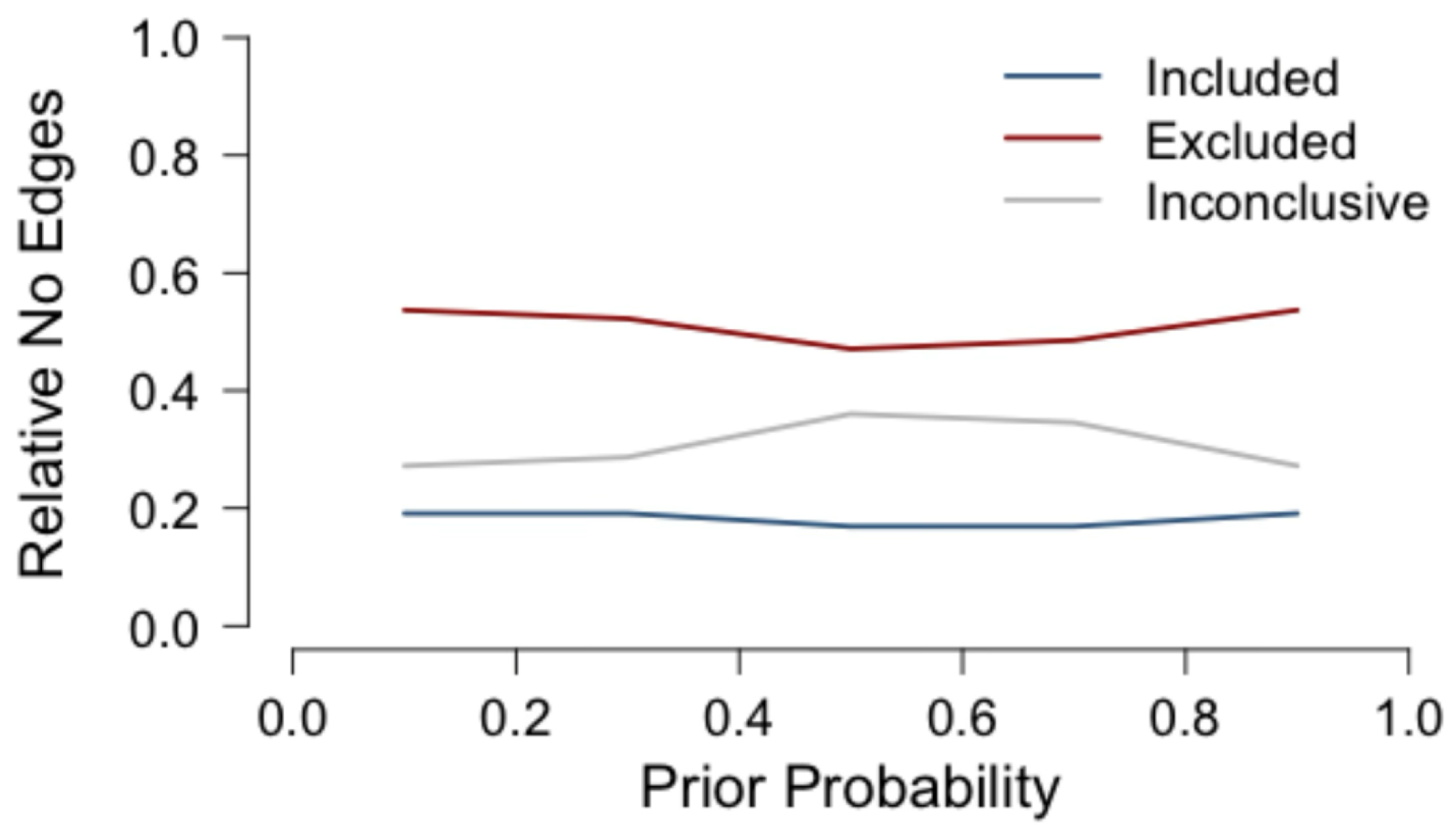
Depicts the results of our prior sensitivity analyses across five different probabilities for edge inclusion priors. Blue depicts the relative number of included edges, red depicts the relative number of excluded edges, and gray represents the relative number of inconclusive edges.

We conducted prior sensitivity analyses across five different probabilities for edge inclusion priors 0.1, 0.3, 0.5, 0.7, and 0.9. The results of the analysis are presented in Figure 8. The networks estimated with various priors showed approximately the same proportion of edges across the three edge inclusion categories, Included, Excluded, and Inconclusive. As expected, a uniform prior with a prior inclusion probability of 0.5 results in the most edges being Inconclusive; the shift across the different inclusion probabilities is mostly in which edges are moved from the Inconclusive category to the Excluded category. The number of Included edges remained stable across the different priors, suggesting that the edges that were included are stable across iterations.

## 4. Discussion

This study is the first to apply a psychometric network approach to investigate the relationships between language practices, bicultural identity, and cognitive functioning. Rather than testing a categorical bilingual vs monolingual advantage, we ask which dimensions of bilingual experience are most associated with attentional control, an approach aimed at clarifying why findings on the bilingual advantage are often mixed. Consistent with accounts that emphasize heterogeneity in bilingual experience (Bialystok & Craik, 2022; Green, 2018; Green & Abutalebi, 2013; Muysken, 2013; Titone & Tiv, 2022), we examine how within-group heterogeneity in bilingual language practices and identity management variables relate to attentional control outcomes, rather than treating bilingualism as a unitary construct.

We purposefully recruited US-born Spanish–English bilinguals of Latin American descent to better isolate the unique relationship between bilingual experience and attentional control that may be obscured in more heterogeneous samples, holding constant key sources of variability that often cloud bilingualism research and contribute to inconsistent estimates of bilingual advantage across studies: language distance, immigration history, and the sociocultural context (de Bruin et al., 2015; Muysken, 2000; K. R. Paap et al., 2015).

By narrowing our inclusion criteria to a single language pair, we account for variations in language distance, or the extent to which two languages overlap in their lexicon (words themselves), morpho-syntax (how the words are constructed and combined), semantics/pragmatics (how meaning is organized and how speakers use the language), and phonology (how similar are their phonemes) (Muysken, 2013), so that the effects in our network could be attributed more confidently to how participants navigate and manage two languages or cultures, rather than to structural differences between the languages they spoke. This, in turn, allows a clearer interpretation of which bilingual experience factors show the most direct associations with attentional control (e.g., language mixing frequency) after accounting for the influence of all other variables in the network.

### 4.1. Network Approaches

One of the strongest use cases of psychometric network analyses is that they enable researchers to conceptualize psychological phenomena as a complex system (Borsboom et al., 2021). This enables us to use the tools of systems and network science to explore the complex ways in which our variables of interest interact with one another across the network (Isvoranu et al., 2022). In accordance with the reporting standards for network psychometrics, we refrain from making strong inferences based on the network analyses alone (Burger et al., 2023).

Importantly, our use of a Bayesian psychometric network approach allows us to determine the relative uncertainty of the network structure as a whole and that of the individual edges (Huth et al., 2023). Our analyses determined that there is considerable uncertainty regarding the most probable network structure, meaning that there were several plausible structures that could have produced our data. At the same time, we are confident about the overall complexity of the underlying network being between 40 and 46 edges.

Based on the independent inspection of any one of these indices, one might decide that an entirely different set of variables are promising candidates for future research. However, the interpretation of network measures should be carefully considered within the context of the study and what the centrality indices purport to measure (Bringmann et al., 2019; Fried, 2018). Even though 24 edges exceeded our evidentiary threshold of a Bayes Factor greater than 10, these edges were primarily concentrated among conceptually related variables, suggesting stronger within-domain organization than widespread cross-domain connectivity. Because the Bayesian model averaging results indicated substantial uncertainty regarding the single most probable network structure, we will focus our interpretation on two edges with the strongest evidence for their existence that also served as bridges between domains: the bridge between cognition and language, Mix-Flanker (${BF}_{10}=49$), and the bridge between identity and language, BSWQ_US-Biis_Harmony (${BF}_{10}=99$).

We used three network measures to investigate the degree of influence variables had within the network, predictability, strength centrality, and bridge strength centrality. To demonstrate the utility of these various indices and the importance of considering multiple network measures, we will look at the unintended switches node (BSWQ_US). Looking at how the various network measures index unintended switches in terms of influence helps us gain broader insight into their role.

In terms of predictability, 39.9% of the variance in unintended switching can be predicted by all the other nodes in the network. Since our estimated network was composed of cross-sectional data, we can think of our estimated predictability as the upper bound. In practice, this means that the true predictability is lower, since we cannot account for the directionality of edges (Haslbeck & Waldorp, 2018). The nodes in our network varied in their Bayesian R^2^ predictability, ranging from 15 to 43% in the extent to which they could be predicted by all other nodes. As a network measure, node predictability is of practical importance since it (a) tells us how strongly a given node is influenced by its neighbors in the network, and (b) helps identify candidate variables for future studies (Haslbeck & Waldorp, 2018).

Given the large number of nodes indexing various aspects of naturalistic language switching practices—the tendency to switch into English (BSWQ_L2S) or Spanish (BSWQ_L1S), contextual switching (BSWQ_CS), unintended switching (BSWQ_US), childhood exposure to a dense code-switching environment (CDCi), and the weighted frequency of language mixing (Mix)—it may be unsurprising that the predictability of unintended switching is high. The predictability of the highest ranked non-linguistic variable measured was the harmony between their two bicultural identities (Biis_Harmony), or alternatively, the lack of conflict emerging as a result of them. At 39.9% in terms of Bayesian R^2^ predictability, unintended switching still has over 60% of the variance unaccounted for, suggesting that there are relevant factors that are missing in our network model. Future researchers interested in using predictability should consider the number of measures that are indexing a particular domain when deciding the relative importance of a given node.

We can use another network measure, strength centrality, to calculate the overall influence that a node has with its neighbors. In our network, unintentional switching (BSWQ_US) shares an edge with contextual switching (BSWQ_CS), switches into English (BSWQ_L2S), bicultural harmony (BiiS_Harmony), and fluid reasoning (RAPM). We calculate strength centrality by adding up the absolute edge weights for edge weights across models. We find that unintended switching has a strength centrality of 0.5, coming in twelveth (out of 17). Other nodes such as attentional control scores (Flanker = 2.07), the weighted frequency of language mixing (Mix = 1.82), and the tendency to switch into English (BSWQ_L2S = 1.19) have higher levels of strength centrality in our network. So, despite being one of the nodes with the highest predictability (39.9%), unintended switching has a low degree of influence in terms of the weighted edges it shares with other nodes.

If the research question involves determining which nodes have the strongest influence on the nodes around them, then researchers should focus on the nodes with the highest strength centrality. However, researchers should ensure that they do not have multiple nodes indexing the same construct, as this would inflate the centrality estimates of those variables (Christensen et al., 2023).

Importantly, our research is motivated by a desire to determine how bilingualism, biculturalism, and cognition interact with each other. This makes our third network index, bridge strength centrality, particularly important. In contrast to strength centrality, bridge strength centrality only considers the edge weights between nodes that fall into distinct communities or clusters. On this metric, Biis_Harmony (0.50) and unintended switching (0.30) come in at the very top, whereas measures like contextual switching (BSWQ_CS = 0.01) and the age at which they started speaking English (AoCeng = 0.01) have little to no influence outside of their direct communities. We did not expect harmony between cultures and unintended switching (partial correlation = −0.17, ${BF}_{10}=99$) to be the bridge nodes between language use and cultural identity. This motivates the question, what could explain the negative association between bicultural harmony (i.e., a decrease in conflict) and the prevalence of unintended switches?

### 4.2. Interpreting Edges

Since our network is estimated from cross-sectional data, there are several potential interpretations for this edge, and future research would be required to determine which of these potential mechanisms are likely at play. If two nodes (X and Y) are statistically dependent, then either (a) X causes Y, (b) Y causes X, (c) there is a third variable Z that causes both X and Y (Isvoranu et al., 2022; Reichenbach, 1956), or (d) Z is a common effect of X and Y (Isvoranu et al., 2022). Network psychometrics is currently best suited as an exploratory tool for identifying psychological phenomena, a critical function in helping us identify plausible avenues for theory formation (Borsboom, 2022; Borsboom et al., 2021). In line with Titone and Tiv’s (2022) Systems Framework of Bilingualism we explore the following interpretations as plausible future research avenues for “a more nuanced, inclusive, and socially informed scientific understanding of the bilingual experience”.

### 4.3. Cultural Harmony (Conflict) and Unintended Switches

Given the rich amount of literature investigating the acculturative process, researchers should look to studies that investigate identity, cultural stressors, and associated outcomes in their specific population of interest. Carreira and Beeman (2014) interviewed hundreds of Latino students about their language experiences growing up in the U.S. They found that heritage speakers were often shy about or held low self-esteem about their heritage language abilities. Themes that emerged from the interviews such as “The burden of never being good enough in Spanish,” “The burden of falling inbetween languages,” and “The burden of being linguistic mestizos” were often related to concerns regarding their ethnic identity and the general status of Spanish in the U.S.

For some of our participants, it is possible that unintentional code-switching could directly result in increased cultural conflict. Their investigation of language attitudes across two Texas border towns Rangel et al. (2015) found that Spanish–English bilingual men and women held negative attitudes towards speakers who code-switched between English and Spanish. In this kind of sociolinguistic environment, code-switching could result in a loss of perceived status and directly drive an increase in conflict. Since heritage language use among heritage bilinguals deviates from the arbitrary standard of how Spanish is spoken by proficient monolinguals, it is often seen as lesser or deficient by members of the ingroup and by scientists studying bilingualism at large (Bayram et al., 2021; Flores & Rosa, 2015). Given the dominance of participants of Mexican and Central American descent in our sample, future work could seek to recruit more individuals with roots in South America and the Caribbean to test whether the observed relationship holds in sociolinguistic environments where code-switching is more socially accepted (Kutlu & Kircher, 2021; Poplack, 1988).

Conversely, it is possible that increased bicultural conflict could result in more unintentional switches. Unintentional switching among balanced bilinguals is conceptualized as originating from a momentary lapse in language monitoring (Green, 1986; Green & Wei, 2014). Prior research has found that the cognitive demands of a task, the overall availability of cognitive resources, and attitudes towards code-switching all affect the prevalence of code-switching (Liu et al., 2024; A. Williams et al., 2020). A potential mechanism for this has been identified across several studies. Matras (2000) found that bilingual speakers can reduce the mental effort required for speech production by removing the language-specific alternatives available to them and essentially automating the choice of expression. A lower switching threshold between the two activated languages has been associated with an increased rate of intended and unintended language switches (X. Li et al., 2021; Zhu et al., 2022). On the cultural conflict side of the edge, researchers have also shown that performance on cognitive tasks can be impacted by stressors like stereotype threat and perceived discrimination via the effects of psychological and biological stress responses (Levy et al., 2016; Rea-Sandin et al., 2021).

Researchers investigating the association between cultural conflict and cognitive performance would benefit from using more nuanced, context-sensitive measures of identity-based cultural conflict. For example, Zeledon et al. (2024) identified location-specific differences (Los Angeles vs. Miami) in three types of cultural stressors among Hispanic/Latinx adolescents: sociopolitical stressors, intragroup marginalization, and language brokering experiences. With regards to sociopolitical stressors, participants in Miami reported increasing stress due to changes in the political administration, whereas participants in Los Angeles reported the opposite. The source of intragroup marginalization was particularly important, with respondents from both groups identifying marginalization coming from family members as more impactful than marginalization coming from peers or others.

Mixed cognitive and psychosocial effects have been observed as a result of language brokering, i.e., the childhood experience of translating or interpreting for family members and others (Morales & Hanson, 2005). Childhood language brokering experience has been identified as a risk factor for increased depression and anxiety (Rainey et al., 2014). For others, Language brokering serves as a protective factor associated with resilience to negative psychosocial outcomes (López, 2020). We will further address language brokering experience since this seems like a promising variable to consider in future research.

Other variables could be playing a role in this relationship as well. Yeh and Lim (2021) found that when bilinguals lived in a monolingual environment, the prevalence of their unintended switches was dependent on their awareness of the linguistic diversity in their environment and their level of executive control. They found that for individuals with high executive control, high awareness of the language environment was associated with lower rates of unintentional switching, whereas the rates of unintended switching were independent of awareness of the language environment among individuals with low executive control. This suggests that some individuals are able to adapt their level of language control, and that this ability is affected by individual differences in cognition.

Prior work has consistently found a negative relationship between attentional control and unintended switches (Festman, 2012; López-Penadés et al., 2020; Rodriguez-Fornells et al., 2012; Soveri et al., 2011). Surprisingly, we did not find evidence for this relationship in our network. We believe that language brokering experiences could help shed light on the relationship between bicultural conflict and unintended switches, as well as the missing edge with attentional control. S. Y. Kim et al. (2024) investigated the relationship between language brokering experiences, discriminatory experiences, and cognitive control. They found that brokering experiences moderated the relationship between discriminatory experiences and their cognitive control performance. Typically, participants with brokering experience showed a negative correlation between discriminatory experiences and cognitive control, however among participants with positive subjective brokering experiences who viewed their brokering experiences as more central to their own identity, this relationship was reversed. This highlights the importance of situating the navigation of multiple languages within the sociocultural context that it is occurring in.

### 4.4. Bilingualism and Attentional Control

One of the central goals in this work is to identify which aspects of navigating multiple languages and cultures have the strongest, most robust, and most direct association with cognition. In our network approach, evidence for or against a bilingual advantage is quantified as conditional associations between specific language use practices and attentional control after accounting for all of the other linguistic or cultural variables in the network. The edge between language mixing therefore speaks directly to Green and Abutalebi’s (2013) Adaptive Control Hypothesis (ACH), in which cognitive control processes beyond inhibition are required to speak in one language over another (e.g., goal maintenance, conflict monitoring, and interference suppression). According to the ACH, the cognitive effects of bilingualism are primarily determined by how and when bilinguals use different languages across the various contexts of their life. Our findings provide partial support for the predictions outlined in the ACH (Green & Abutalebi, 2013). The ACH specifies three distinct interactional contexts, a single-language context (SLC), dual-language context (DLC), and a dense code-switching context (DCS). In a SLC (e.g., Spanish exclusively used at home, and English exclusively used at work/school), the context itself functions as a cue for the appropriate language. As a result, the researchers hypothesize that this pattern of language use trains the use of attentional control to maintain the current language while monitoring for conflict and preventing interference from the language they are not currently using.

In our sample of heritage Spanish–English bilinguals, after accounting for the relationships among all other variables in the network, we find that the more participants reported mixing languages in normal conversation with friends, family, classmates, and others (i.e., the less time they spent in a single-language context, SLC), the lower they scored on the Flanker task. Specifically, we found strong evidence (${BF}_{10}=49$) for a weak negative edge weight (partial correlation = −0.15) in the network connecting language mixing frequency and attentional control, as measured by the Flanker task. This pattern of results is consistent with experience-dependent accounts of bilingualism (Bialystok & Craik, 2022; Green, 2018; Green & Abutalebi, 2013; Muysken, 2013; Titone & Tiv, 2022) and provides strong evidence that the associations between bilingualism and attentional control are not uniform in our sample of heritage Spanish–English bilinguals. Instead, they depend on the prevalence of an individual’s interactional contexts and the language use demands they experience in them.

We did not find support for the notion that time spent in a dual-language context (DLC) would also train the same proactive attentional control processes as a SLC. The ACH (Green & Abutalebi, 2013) stipulates that individuals who use different languages within the same context (a dual-language context, DLC) need to change their languages to match their current conversational partner (e.g., needing to speak Spanish with coworker 1 and English with coworker 2). Therefore, a DLC context would train an individual’s ability to monitor their environment for salient cues that aid them in selecting the appropriate language in addition to training the same proactive control processes as a SLC.

It is possible that the ACH’s focus on the environmental context of the DLC rather than the interlocutor conflates two different sets of environmental conditions with their own respective cognitive demands, switching between languages in a single location and inter-sentential switching between languages in a conversation. It may be that different attentional mechanisms are trained within each of these; for example, if you need to change your language to match your conversational partner, then the context of who you are speaking with mimics the conditions found in a SLC, where attentional control would be trained in order to maintain the current language while monitoring for conflict and preventing interference from the language they are not currently using.

Alternatively, suppose your conversational partner is bilingual and participants switch between but not within utterances. In this case, both languages are at play in a conversation. This would penalize the gating of a single language and instead train an individual’s ability to monitor their conversational partner for salient sociolinguistic cues that aid them in selecting the appropriate language. Support for this interpretation can be found in Dagmar Scheu (2000), where they studied the cultural constraints on code-switching in a bilingual German–Spanish school and found that inter-sentential switching is primarily based on shifts to the topic of conversation and culturally loaded switches. Within their school context, German was associated with academics, whereas Spanish was used in informal communication among students. For these individuals in what would have been considered a DLC, the language of choice was determined by the topic and the purpose of communication. Importantly, the social context of the language environment accepted and embraced the use of both languages, functioning as a sign of their membership in the bilingual community.

Researchers have proposed that individuals are more likely to show transfer from (linguistic) experience to cognitive tasks when the demands of the task itself more closely reflect the demands of the individual’s (linguistic) experiences (Y. Kim et al., 2023; von Bastian et al., 2020). We believe that the specific demands found in the Flanker task are aligned with the kinds of demands present in a SLC (Green, 1998; Green & Abutalebi, 2013). The Flanker task requires selective attention; in it, you have to attend to the centrally presented stimulus and ignore the flanking stimuli around it (Diamond, 2013). Framed another way, this is indexing an individual’s ability to avoid distraction emerging from the perceived environment (von Bastian et al., 2020). This narrowing of attention mimics the kind of attentional shift that is linked to the demands of a SLC environment, where you have to enhance the mental representations of the language you are currently using in order to minimize the influence of the language that is not being actively used (Green & Abutalebi, 2013).

Conversely, narrowing of attention comes at a cost if you need to pay attention to the environment for sociolinguistic cues (Del Giudice & Crespi, 2018; Leivada et al., 2023). In Eriksen and St. James’s (1986) zoom model of attention, a tradeoff exists between the breadth and resolution of the activated mental representation. Leivada et al. (2023) proposes we view the cognitive effects of bilingualism through the lens of tradeoffs. They argue that without considering the positive and negative consequences of adaptation, we are only able to see half of the overall picture. They propose a systematic investigation across domain-specific pairings to help determine the unique neurocognitive tradeoffs that emerge from linguistic adaptation. Future researchers should utilize this framework of tradeoffs and include tasks that are designed to capture the flexibility and/or breadth of attention, as they should be better indices of the kinds of tradeoffs that might be observed in code-switching environments (Leivada et al., 2023).

### 4.5. Limitations and Future Directions

Gaussian copula graphical models, GCGMs, are undirected, which comes with pros and cons. They require fewer observations, can accommodate cyclical networks, and do not require prior knowledge of the underlying structure (Isvoranu et al., 2022). Due to the undirected nature of the network, the partial correlations do not represent causal relationships. We cannot know for sure whether (a) more frequent mixing of languages during a typical day causes lower scores on the attentional control Flanker task, (b) whether higher levels of conflict arising from managing two distinct cultural identities cause individuals to unintendedly switch between their languages, or (c) if some other factor not captured in our network is the culprit. However, these undirected networks can serve as a starting point for future researchers looking to investigate causal mechanisms using longitudinal network modeling (Borsboom, 2022; Goulter et al., 2022).

In estimating our GCGM, we used a default prior where every edge had a 50% chance of inclusion. Although we conducted prior sensitivity analyses to ensure the robustness of our findings, future work could utilize prior literature on the population under study to implement informed priors for variable pairs whose associations are known (e.g., the relationship between the BIIS-2 and the MISS in people of Latin American descent in the United States). Using informed priors could lead to a conditional dependence structure that more accurately reflects what is known about the variables under examination.

Since our entire study was conducted in English, it is possible that the language of assessment could be considered a limitation. Language may act as a prime in bilinguals, influencing which cultural frames or response tendencies are most accessible or deployed (Adler et al., 2020; Carbajal et al., 2023; Zhu et al., 2022). So, for English–Spanish heritage speakers, an English-only administration may have privileged English-only experiences, primed a SLC control mode, or affected responses on measures related to language use, bicultural identity, and memory-based self reporting. It is therefore possible that a Spanish-language or mixed-language administration could have produced a somewhat different pattern of association among the variables. Future work should directly compare English and Spanish administration, or include bilingual assessment designs, to test whether the language of assessment systematically alters these relationships.

We agree with Titone and Tiv (2022) that researchers need to expand the scope of their investigations and account for the sociocultural context in which language use and cognition are being shaped. For heritage Spanish–English speakers in the US in particular, it seems that the addition of measures indexing language brokering experiences and their subjective attitudes towards them, as well as towards the language as a whole, would provide crucial insight into complex ways these variables interact. Future researchers looking to implement a socially infused approach to bilingualism will need to expand their code-switching measures to account for the specific types of switching practices that are common in bilingual communities around the world (Muysken, 2013) in addition to the socio-affective components present in that environment. The code-switching measures we used in our study focused on the distinctions proposed by Green and their colleagues regarding interactional environments, as well as inter-sentential and intra-sentential switches (Green & Abutalebi, 2013; Green & Wei, 2014). Unfortunately, these measures do not distinguish between different types of intra-sentential switches such as insertion, alternation, congruent lexicalization, and back-flagging, despite their varied social (Muysken, 2013) and cognitive (Green, 2018) implications.

An important limitation is that our exploratory network was estimated on a population of heritage Spanish–English speakers that identified as both bicultural and proficient balanced bilinguals. This means that our observed pattern of relationships should only be expected to generalize to a similar population. Researchers who are interested in understanding the relationship between heritage identity, heritage language use, and the social environment that shapes them should consider extending the population of interest to include individuals with limited Spanish language proficiency. Latinés with limited heritage language proficiency demonstrate deep cultural ties to their heritage, while their lack of Spanish proficiency introduces barriers to full acceptance and belonging within their own cultural communities (Callesano, 2023; Stransky et al., 2022).

By including a broader spectrum of heritage bilinguals, it is likely that nodes that played a limited role in our network (i.e., Spanish language proficiency) would play a much stronger role in moderating the structure of the resulting network. Prior work investigating how this group navigates the intersection of language and culture has primarily been qualitative, which, while providing rich narratives, limits the ability to generalize findings across the broader Latiné population in the United States (Stransky et al., 2022; Tseng, 2021). To bridge this gap, research could benefit from incorporating quantitative methods that allow for the measurement and analysis of these experiences across larger samples. By doing so, scholars can enhance our understanding of the complex dynamics at play, providing a more comprehensive picture of how language proficiency, or the lack thereof, influences cultural identity and cognitive development on a broader scale.

We believe that our work represents an important step towards understanding the complex pattern of interactions between language, identity, and cognition. The use of new paradigms or methods such as network approaches allows us to move beyond trying to predict a single variable towards investigating complex phenomena by considering the reciprocal interactions between every variable under consideration (Borsboom et al., 2021). Psychometric network analyses can also be used to compare the pattern of conditional associations between populations that vary on variables of interest, helping grow our understanding of the context-specific aspects of bilingualism. Using exploratory psychometric network analysis, we also identified aspects of bilingualism that serve as bridges to the domains of cognition and identity, identifying promising avenues for future research. In line with theories of complex systems, it is only by considering the interplay between cognition and the linguistic/cultural demands that individuals experience in their everyday lives that we can begin to get a sense of how experience-dependent changes manifest (Bronfenbrenner, 1977; Munakata et al., 2023; Titone & Tiv, 2022).

## 5. Conclusions

For researchers looking to identify the circumstances under which a bilingual advantage may be present, a network approach provides a methodological advantage over traditional regression or ANOVA-based analyses that are common in bilingualism research. Modeling conditional dependencies among sociocultural, linguistic, and cognitive variables provides a more holistic view of the complex system underlying bilingualism, biculturalism, and cognition. This approach is more in line with emergentist approaches (Bates, 1976; Bronfenbrenner & Ceci, 1994) and recent theoretical developments that conceptualize bilingualism as an emergent property of dynamic interactions between person-specific factors and factors specific to the sociolinguistic environment (Titone & Tiv, 2022).

Our findings contribute to a growing body of literature suggesting that the effects of managing two languages or cultures on cognition are context-specific, meaning that these adaptations emerge as a response to the specific cultural and linguistic demands in their social environment (Bialystok, 2017; Green & Abutalebi, 2013; Tadmor & Tetlock, 2006; Woumans & Duyck, 2015; Xie et al., 2022). Importantly, our Bayesian psychometric network approach allowed us to estimate the uncertainty associated with the edges we found in addition to quantifying the evidence for missing edges. By identifying nodes that have a greater degree of influence on the broader network and mapping the connections and lack thereof among measures indexing language, bicultural identity, and cognition, we offer a more holistic, socially infused characterization of how bilingualism, biculturalism, and cognition interact in heritage speakers of Spanish in the United States.

## Figures and Tables

**Table 1:** Sociodemographic characteristics of participants.

Baseline Characteristic	n	%
Gender		
Woman	207	51.2
Man	178	44.1
Non-binary	18	4.5
Missing	1	0.2
Ethnicity		
Latiné/Hispanic	378	93.6
Black, Latiné/Hispanic	12	3.0
White	5	1.2
Indigenous/Native	4	1.0
An ethnic group not mentioned above	2	0.5
African	1	0.2
Asian	1	0.2
Missing	1	0.2
Generational status		
2nd generation	327	80.9
3rd generation	48	11.9
4th generation	20	5.0
1st generation	7	1.7
1.5 generation	2	0.5
Family income		
Less than $10,000	18	4.5
$10,000–$19,999	29	7.2
$20,000–$29,999	33	8.2
$30,000–$39,999	51	12.6
$40,000–$49,999	43	10.6
$50,000–$59,999	54	13.4
$60,000–$69,999	29	7.2
$70,000–$79,999	32	7.9
$80,000–$89,999	22	5.4
$90,000–$99,999	25	6.2
$100,000–$149,999	47	11.6
More than $150,000	21	5.2
Race		
White	268	66.3
Racial group(s) not mentioned above	99	24.5
Native American/American Indian/First Nation	47	11.6
Black	25	6.2
Asian	8	2.0
Middle Eastern/North African	3	0.7
Alaskan Native	1	0.2
Missing	9	2.2
Selected Multiple Racial Identities	45	11.1

Note: n=404. Participants were on average 30.3 years old (SD=9.3).

**Table 2:** Diagonal weights matrix for variables included in the network.

Variable	1	2	3	4	5	6	7	8	9	10	11	12	13	14	15	16
1. Profspa																
2. AoCspa	−0.10															
3. AoCeng	0.01	0.29														
4. Mix	0.00	0.07	−0.03													
5. Usespa	0.13	−0.14	0.35	0.20												
6. BSWQ_L1S	−0.32	0.00	0.00	0.00	−0.16											
7. BSWQ_L2S	0.18	−0.01	0.00	0.01	0.12	0.24										
8. BSWQ_CS	0.01	0.00	0.00	0.30	0.00	0.16	0.31									
9. BSWQ_US	0.00	0.00	0.00	0.00	0.01	0.00	0.16	0.26								
10. CDLi	0.00	−0.01	0.00	0.10	0.02	0.00	0.09	0.00	0.10							
11. CDCi	0.00	−0.16	0.00	0.20	0.01	0.00	0.00	0.21	0.00	−0.20						
12. MISS_Hybrid	0.00	0.01	0.00	0.00	0.00	0.00	−0.01	0.00	0.01	0.00	0.00					
13. MISS_Alternating	0.06	0.00	0.00	0.00	0.02	0.00	0.00	0.00	0.00	−0.01	0.00	0.32				
14. Biis_Harmony	0.02	0.00	0.00	0.01	0.00	−0.02	−0.06	0.00	−0.18	0.08	0.00	0.32	−0.44			
15. RAPM_score	0.00	−0.06	0.00	0.00	0.00	0.00	0.00	0.00	−0.09	0.00	0.00	−0.01	0.00	−0.04		
16. Stroop	0.01	0.00	0.00	0.00	0.00	0.00	0.01	0.00	−0.02	−0.03	0.07	0.00	−0.01	0.09	0.36	
17. Flanker	0.00	−0.03	0.00	−0.15	0.00	0.00	0.00	−0.01	−0.02	0.00	0.00	0.01	0.01	0.00	0.41	0.48

Note: Values represent partial correlations. Only the lower triangular matrix is displayed.

**Table 3:** Bayesian model comparison for the top 10 network structures.

Model Rank	*p*(*H*|*Data*)	${\mathrm{BF}}_{\mathrm{tj}}$
1	$1.74\times 10^{-3}$	1.00
2	$1.22\times 10^{-3}$	1.43
3	$1.14\times 10^{-3}$	1.53
4	$1.14\times 10^{-3}$	1.53
5	$1.10\times 10^{-3}$	1.58
6	$1.10\times 10^{-3}$	1.58
7	$1.10\times 10^{-3}$	1.58
8	$1.08\times 10^{-3}$	1.61
9	$1.02\times 10^{-3}$	1.71
10	$9.20\times 10^{-4}$	1.89

Shown are the posterior model probabilities as well as the Bayes factors indicating how much better the top model is at describing the data than the *j*th model.

**Table 4:** Network metrics.

Node	Strength	Bridge Strength	Predictability
Profspa	0.90	0.09	0.15
AoCspa	1.09	0.10	0.29
AoCeng	1.04	0.01	0.29
Mix	1.82	0.16	0.33
Usesspa	0.88	0.03	0.31
BSWQ_L1S	0.47	0.02	0.35
BSWQ_L2S	1.19	0.08	0.43
BSWQ_CS	0.21	0.01	0.43
BSWQ_US	0.50	0.30	0.40
CDLi	0.21	0.12	0.20
CDCi	0.85	0.08	0.33
MISS_Hybrid	0.60	0.06	0.17
MISS_Alternating	0.44	0.11	0.31
Biis_Harmony	1.03	0.50	0.33
RAPM_score	1.15	0.21	0.24
Flanker	2.07	0.24	0.32
Stroop	0.80	0.23	0.30

Note: Strength centrality and predictability are calculated using the Bayesian model averages in order to determine uncertainty. Bridge strength is a point estimate calculated from the most likely model.

## Data Availability

The data presented in this study are available from the corresponding author upon request. The data are not publicly available due to privacy and Institutional Review Board restrictions; all shared data will be de-identified.
